# Synthesis of New Asymmetrical Chalcones and Evaluation of Their Use in Combination with Curcumin Against Rhodesain of *T. brucei rhodesiense*

**DOI:** 10.3390/ijms27073320

**Published:** 2026-04-07

**Authors:** Carla Di Chio, Josè Starvaggi, Benito Natale, Santo Previti, Fabiola De Luca, Sandro Cosconati, Tanja Schirmeister, Maria Zappalà, Roberta Ettari

**Affiliations:** 1 Department of Chemical, Biological, Pharmaceutical and Environmental Sciences, University of Messina, Viale Ferdinando Stagno d’Alcontres 31, 98166 Messina, Italy; cdichio@unime.it (C.D.C.); jose.starvaggi@studenti.unime.it (J.S.); spreviti@unime.it (S.P.); fabiola.deluca@unime.it (F.D.L.); mzappala@unime.it (M.Z.); 2 Department of Environmental, Biological and Pharmaceutical Sciences and Technologies DiSTABiF, University of Campania Luigi Vanvitelli, Via Vivaldi 43, 81100 Caserta, Italy; benito.natale@unicampania.it (B.N.); sandro.cosconati@unicampania.it (S.C.); 3 Institute of Pharmacy and Biochemistry, University of Mainz, Staudingerweg 5, 55128 Mainz, Germany; schirmei@uni-mainz.de

**Keywords:** combination study, nutraceutical, curcumin, cysteine protease, synergistic effect

## Abstract

Rhodesain is a cysteine protease that plays a key role in the life cycle of *Trypanosoma brucei rhodesiense*, an endemic parasite in sub-Saharan Africa and responsible for Human African Trypanosomiasis (HAT), a disease that can be fatal if not treated promptly. Due to the limitations associated with current HAT pharmacological therapy, the search for new targets for the development of antitrypanosomal agents is urgently needed; in this context, rhodesain represents a promising therapeutic target. In this study, new chalcones were synthesized and tested against rhodesain. Given their affinity for the trypanosomal cysteine protease (*K*_i_ values in the micromolar range), chalcone **1a** was selected to evaluate its effect in combination with the nutraceutical curcumin. The Combination Index (CI) was calculated using Chou and Talalay’s method. The analysis of the CI calculated at different f_a_ values of enzyme inhibition for the combination curcumin + **1a** showed promising results. For all f_a_ values, the CI is less than one, indicating a synergistic effect when chalcone **1a** is combined with curcumin. In particular, at the most significant f_a_ value (0.90), corresponding to 90% of enzyme inhibition, the CI value is 0.1781, indicating a strong synergism between the synthetic drug and the nutraceutical. The combined use of curcumin and chalcone **1a** led to an enhancement of rhodesain inhibitory activity, resulting in a strong synergistic effect and supporting further investigation of this combination.

## 1. Introduction

Human African Trypanosomiasis (HAT), also known as sleeping sickness, is classified as a Neglected Tropical Disease (NTD) [1]. It is an endemic disease prevalent in sub-Saharan Africa where socioeconomic and living conditions favor its persistence. The infection is caused by protozoa belonging to the genus *Trypanosoma*, which is transmitted to humans via the bite of a tsetse fly (genus *Glossina*). The subspecies *T. brucei gambiense* is responsible for the chronic form of the disease and is widely distributed in western and central Africa; while *T. brucei rhodesiense* causes the acute form, which occurs mainly in eastern and southern Africa [2].

The clinical features of HAT depend on both the infecting *Trypanosoma* subspecies and the disease stage. It is often misdiagnosed both in non-endemic areas, where the disease is poorly recognized, and in endemic regions, where it lacks specific symptoms and is commonly confused with malaria [3,4]. Trypanosomiasis progresses through two stages; during the haemolymphatic stage, the parasites disseminate via the bloodstream and lymphatic system, producing non-specific symptoms such as fever and generalized weakness. When the parasites cross the blood–brain barrier, the disease advances to the neurological stage, characterized by sleep–wake cycle disturbances and progressive neuropsychiatric impairment, which may ultimately lead to coma and death if left untreated [5].

Only a limited number of drugs are available for the treatment of this infection, and their selection depends on several factors, including the stage of the disease, as well as the patient’s age, body weight, and the infecting species. In the early stage, pentamidine and suramin are commonly used, whereas the second stage is treated with melarsoprol, eflornithine, or nifurtimox. Notably, nifurtimox is recommended in combination with eflornithine (NECT) for managing the second stage of the gambiense form [6]. In 2019, the European Medicines Agency approved fexinidazole, a 5-nitroimidazole derivative, for the treatment of gambiense human African trypanosomiasis. This drug represents a significant advancement, as it is the first orally administered therapy for this condition [7]. More recently, an open-label study was undertaken to assess the efficacy and safety of fexinidazole in the treatment of the rhodesiense form. The World Health Organisation (WHO) has broadened the therapeutic indication for fexinidazole to encompass the trypanosomiasis caused by *T. brucei rhodesiense*. However, owing to the limited availability of clinical data, current recommendations are supported by very low certainty evidence. Furthermore, according to the treatment eligibility criteria, pediatric patients and women in the first trimester of pregnancy are excluded from receiving fexinidazole [8].

Another candidate oral drug is acoziborole (SCYX-7158), an oxaborole-6-carboxamide, which is currently undergoing clinical evaluation. Owing to its long half-life, it can be administered as a single-dose regimen, which may significantly improve treatment adherence. Acoziborole may be considered an excellent therapeutic alternative for the management of both the first and the second stages of trypanosomiasis [9,10].

The limited number of drugs available for treating sleeping sickness, coupled with issues such as toxicity, challenging routes of administration, and emerging drug resistance, highlights the urgent need to discover novel antitrypanosomal targets. In this context, rhodesain, a cysteine protease of *T. brucei rhodesiense*, emerges as a particularly promising target; also known as cathepsin L-like protease, it belongs to clan CA, family C1. The enzyme consists of a single polypeptide chain of 215 amino acids, organized into two distinct domains, known as the left (*L*) and right (*R*) domains. Its catalytic triad, Cys25, His162, and Asn182, is located within a cleft formed between these domains [11].

Rhodesain is essential for multiple biological processes of the parasite, including the evasion of the host immune response and degradation of both intracellularly transported host proteins and parasite-derived proteins. Furthermore, this enzyme plays a critical role in the neurological stage of trypanosomiasis by enabling the parasite to cross the blood–brain barrier [12,13,14,15]. Consequently, rhodesain represents a promising target for the development of novel inhibitors for HAT treatment.

**Figure 1:**
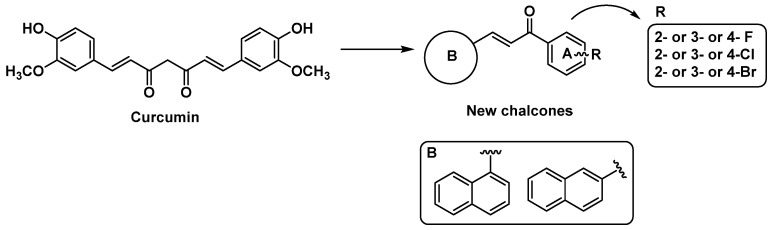
Development of new chalcones starting from curcumin.

In this context, curcumin is a natural polyphenolic compound derived from the rhizome of *Curcuma longa*. It is well known in the literature for its multiple pharmacological activities, as antioxidant, anti-inflammatory, anticancer, neuroprotective, antibacterial and antiparasitic activities [16,17,18,19,20,21,22,23]. It has also been demonstrated to act as a non-competitive inhibitor of rhodesain [24], providing a biologically relevant scaffold for future inhibitor development. As reported in the literature, several naturally occurring compounds have historically served as valuable starting points for the rational design of new bioactive molecules [25,26,27,28,29,30,31]. On this basis, our research group has carried out the synthesis and biological evaluation of a new series of asymmetric chalcones derived from this core structure (Figure 1). Chalcones are structurally similar to curcumin; they possess a carbonyl system in which the diketone has been replaced with an α,β unsaturated system, and the C7 bridge is shortened to improve chemical and metabolic stability [32]. Structure–activity relationship (SAR) studies were conducted on rings A and B to assess how the position and electron-withdrawing properties of substituents influence the inhibitory activity.

In recent years, growing attention has been directed towards the combined use of nutraceuticals and synthetic inhibitors as a promising strategy to enhance the therapeutic efficacy, while minimizing adverse effects and the emergence of resistance for the treatment of various pathologies: inflammation, infectious diseases and cancer [23,33,34,35,36,37].

**Figure 2:**
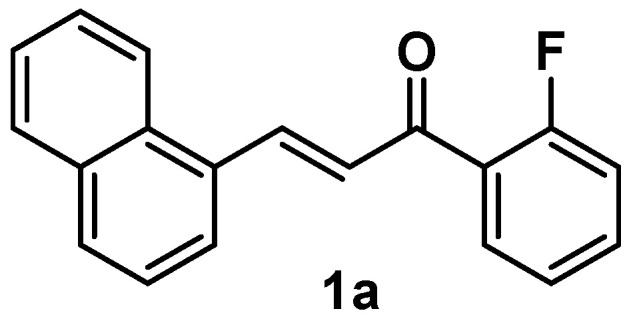
Structure of chalcone **1a**.

Recently, our research group demonstrated the ability of curcumin to improve the pharmacological profiles of synthetic inhibitors if used in combination [24,38,39]. Thus, considering the highest binding affinity (*K*_i_) among all the synthesized compounds as a parameter (see Table 1), it appears that **1a** was a good starting point for the experiments, i.e., for the combination study with curcumin (Figure 2).

In this paper, we now report the synthesis of chalcones **1-2 a-i** and the combination study of the synthetic inhibitor **1a** with curcumin against rhodesain, using the Chou and Talalay method, to establish if the combination has a synergistic, additive or antagonistic effect [40].

## 2. Results and Discussion

### 2.1. Chemistry

**Scheme 1:**
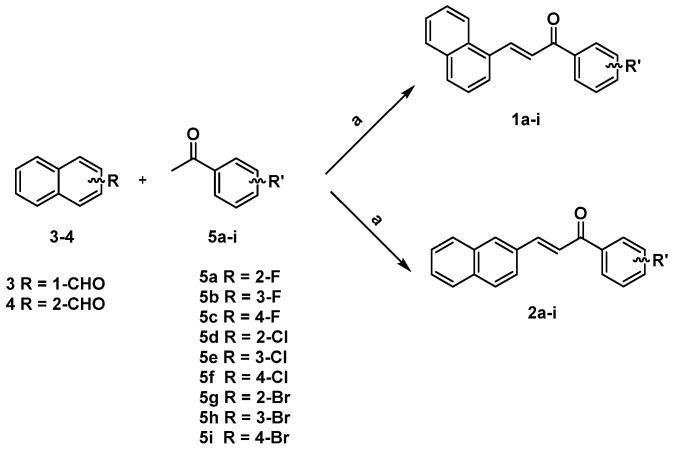
Reagents and condition: (a) EtOH/H_2_O, NaOH, 0 °C then r.t.

The novel chalcones **1-2 a-i** were synthesized, as reported in the literature [41,42,43], using a Claisen Schmidt reaction, between the 1- or 2- naphthaldehyde (**3-4**) and halogen-substituted acetophenones (**5 a-i**) in a hydroalcoholic solution of sodium hydroxide (Scheme 1). The final compounds were obtained in high purity by recrystallization from ethanol or purified by column chromatography, yielding chalcones **1-2 a-i** in good overall yields.

### 2.2. Biological Evaluation

All compounds **1-2 a-i** were tested against rhodesain with fluorometric assays with the specific fluorogenic substrate, Carbobenzyloxy-Phe-Arg 7-amido-4-methylcoumarin hydrochloride (Cbz-Phe-Arg-AMC). A preliminary screening was performed at fixed inhibitor concentration (100 µM) and only compounds that inhibited at least 70% of enzyme activity were further tested at seven different concentrations to determine their dissociation constants (*K*_i_) of each compound, using Equation (1) (see Section 3).

The inhibitory activity of the synthesized compounds (Table 1) was evaluated for series **1**, containing a 1-naphthyl moiety, and series **2**, containing a 2-naphthyl scaffold. Among all tested compounds, **1a**, bearing a 2-fluoro substituent on the 1-naphthyl scaffold, emerged as the most active rhodesain inhibitor, with a *K*_i_ value of 2.00 ± 0.19 µM. Within series **1**, fluorine substitution at the *ortho* or *meta* positions was generally favorable, as illustrated by 3-F (**1b**), which retained a significant potency (*K*_i_ = 2.61 ± 0.02 µM), whereas the *para*-fluoro analogue (**1c**) showed only a moderate inhibition (58% at 100 µM), indicating that *para* substitution reduces the binding affinity. Chloro and bromo substitutions on the 1-naphthyl scaffold were less effective. The 2-Cl (**1d**), 3-Cl (**1e**), and 4-Cl (**1f**) derivatives displayed moderate inhibition ranging from 35% to 50%, while 2-Br (**1g**) displayed a reduced potency (*K*_i_ = 7.16 ± 1.20 µM), and 3-Br (**1h**) and 4-Br (**1i**) achieved only 60% and 40% inhibition, respectively. These observations suggest that steric and electronic effects of the halogen substituents significantly influence the binding, and that the small size of fluorine at the *ortho* position allows optimal interactions within the enzyme active site.

The 2-naphthyl series (series **2**) exhibited notable shifts in activity compared with 1-naphthyl analogues. Fluoro derivatives again displayed a position-dependent potency, with 3-F (**2b**) being the most active in this series (*K*_i_ = 2.52 ± 1.82 µM), while 2-F (**2a**) and 4-F (**2c**) were only moderately active (showing 56% inhibition). Interestingly, *meta*- and *para*-chloro substituted derivatives in the 2-naphthyl series exhibited an improved activity compared with the corresponding 1-naphthyl compounds. For instance, 3-Cl (**2e**) and 4-Cl (**2f**) exhibited *K*_i_ values of 2.93 ± 0.55 µM and 2.54 ± 0.31 µM, respectively, suggesting that the 2-naphthyl orientation better accommodates within the enzyme binding pocket for these substituents. Similarly, 3-Br (**2h**) showed an improved potency (*K*_i_ = 2.95 ± 1.11 µM) compared with its 1-naphthyl analogue (**1h**), whereas 2-Br (**2g**) and 4-Br (**2i**) were only moderately active, underscoring the importance of both substitution position and steric factors.

Comparing the two series, it is evident that the 1-naphthyl scaffold bearing an *ortho*-fluoro group, represented by **1a**, provides the optimal orientation and interactions for maximal rhodesain inhibition. The SAR analysis indicates that the naphthyl position and halogen substitution pattern collectively determine the binding efficiency, with the 2-naphthyl orientation enhancing the potency for certain *meta*- and *para*-substituted chloro and bromo derivatives, although none surpass the activity of **1a**. Overall, these findings highlight the critical role of both scaffold orientation and halogen placement in guiding the design of potent rhodesain inhibitors, positioning **1a** as the benchmark compound for further optimization within this series.

**Figure 3:**
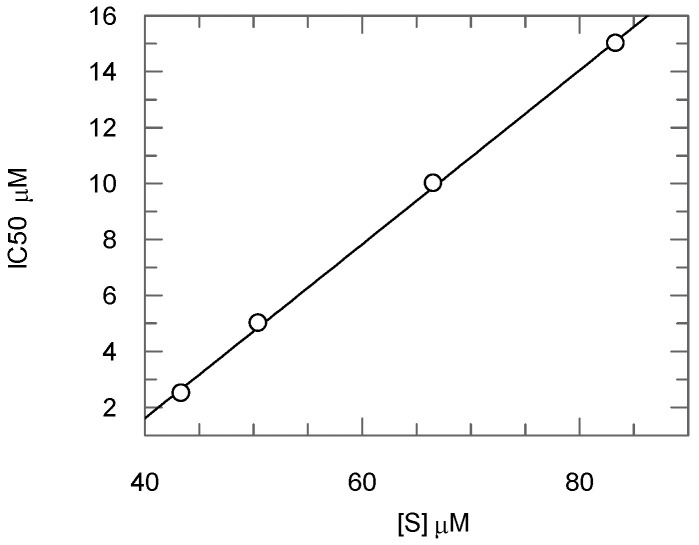
IC_50_ values of inhibition of rhodesain activity in dependence of the substrate concentration (2.5, 5, 10, and 15 μM) for **1a**.

To further investigate if the inhibition is competitive for this class of chalcones, with respect to the substrate, we determined for **1a** the IC_50_ in dependence of four substrate concentrations (2.5, 5, 10, and 15 μM). From this assay (Figure 3), we found linearly increasing IC_50_ values with increasing substrate concentration, demonstrating that chalcone **1a** binds to the active site differently from curcumin, which we previously demonstrated to act as a non-competitive inhibitor [24].

**Figure 4:**
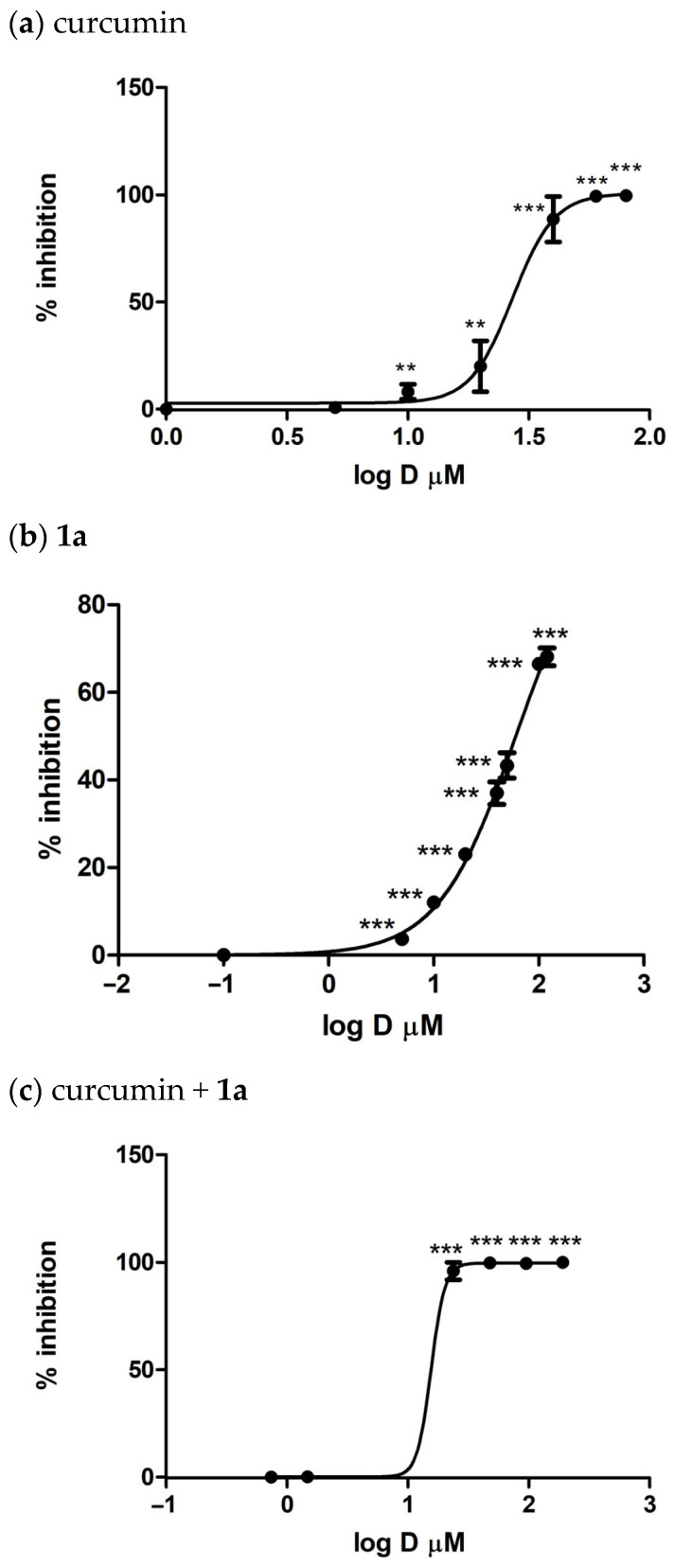
Dose response curves against rhodesain of curcumin (**a**), **1a** (**b**), curcumin + **1a** (**c**). Each experiment was performed in duplicate with ** *p* < 0.01, *** *p* < 0.001 *vs.* no inhibitor.

The experimental protocol for evaluating the combination of the two inhibitors, i.e., curcumin and **1a**, against rhodesain initially involved a preliminary screening at fixed concentrations of each inhibitor (100 μM and 10 μM) to determine the range of activity of the two inhibitors. Subsequently, Tian dilution assays of curcumin and **1a** were performed at seven different concentrations, separately for each inhibitor, in duplicate. The concentrations were selected to cover the range from the minimum enzyme-inhibiting dose to the maximum inhibitory concentration. Curcumin was tested at concentrations ranging from 80 μM to 1 μM, whereas **1a** was tested in a range from 100 μM to 5 μM. Figure 4 shows the dose–response curves of each compound, from which the IC_50_ values were calculated: 25.77 ± 0.19 μM for curcumin and 69.55 ± 0.24 μM for **1a**.

After independently determining the IC_50_ values of curcumin and **1a**, six concentrations (1/128 × IC_50_, 1/64 × IC_50_, 1/4 × IC_50_, 1/2 × IC_50_, IC_50_, and 2 × IC_50_) were selected, as reported in Table 2, to evaluate the effect of their combination. From the resulting dose–response curves (Figure 4c), the IC_50_ value of the combination was then calculated, resulting in 11.18 ± 0.85 μM.

**Figure 5:**
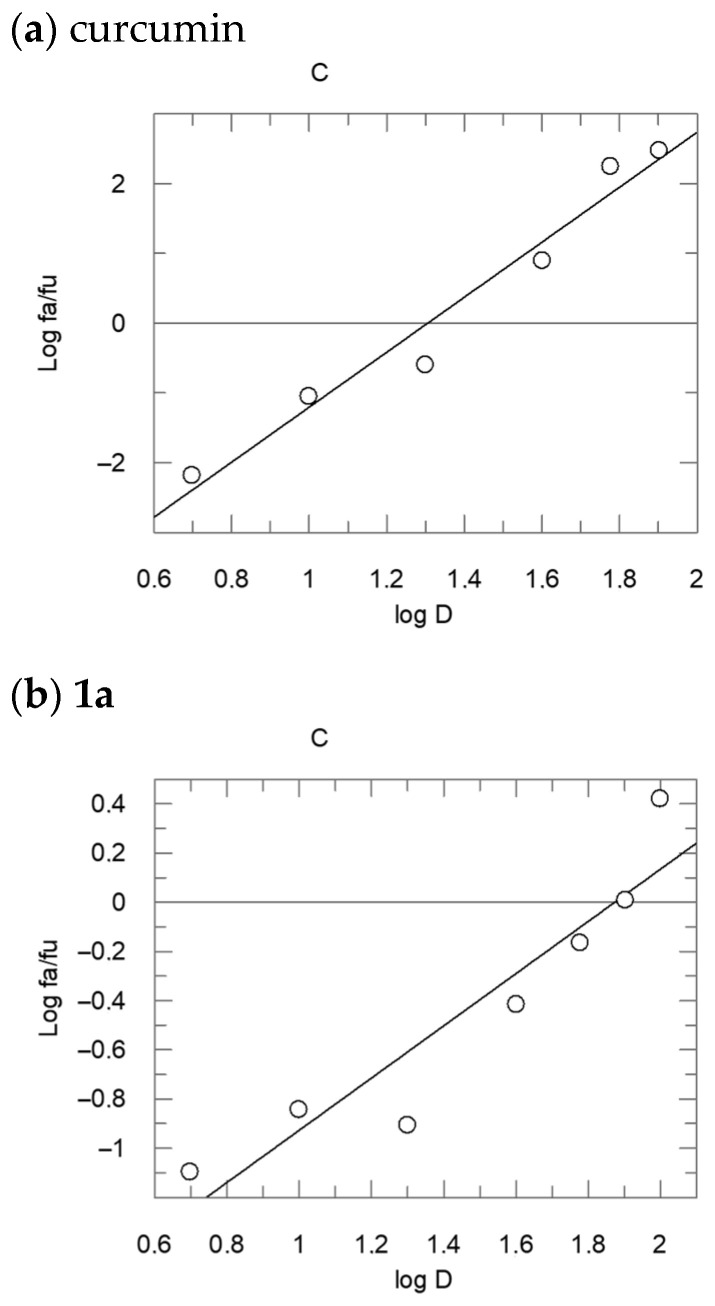

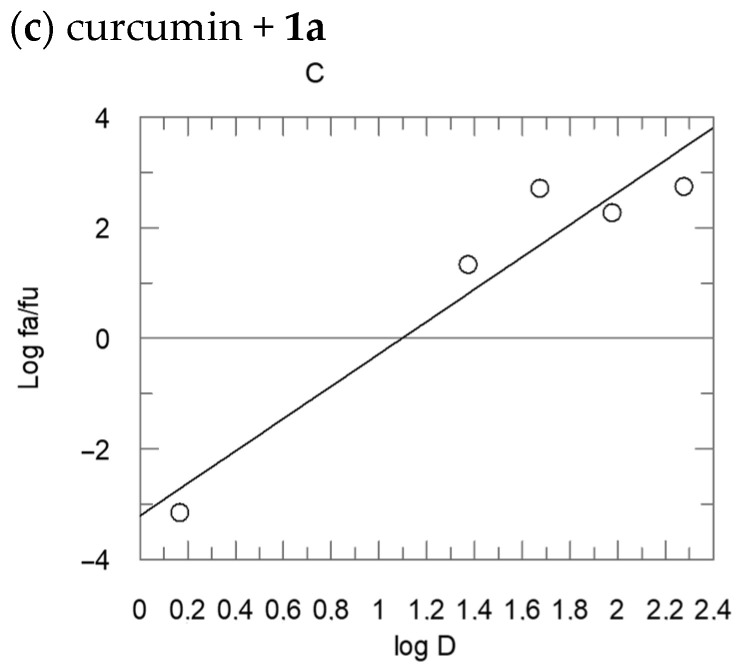
Median Effect Plot for curcumin (**a**), **1a** (**b**), curcumin + **1a** (**c**). D is the dose, f_a_ the affected fraction, and f_u_ the unaffected fraction by rhodesain.

Each dose–response curve was subsequently converted into a Median Effect Plot, where log(f_a_/f_u_) was plotted against log(D) (Figure 5). Unlike the conventional dose–response curve, in which the maximum effect corresponds to 100, this diagram is normalized to 1, as f_a_ + f_u_ = 1. In this context, $f_{a}$ is the fraction of inhibited enzyme, while $f_{u}$ is the fraction that remains active at each dose (D).

Using Grafit software (Version 5.0.1.3; Erithacus Software Limited, East Grinstead, West Sussex, UK), the slope of the straight line, “m” values, of the Median Effect Plots were determined: for curcumin, m_1_ = 3.9427; for **1a**, m_2_ = 1.0620; and for the curcumin–**1a** combination, m_1,2_ = 2.9247 (molar ratio of 0.37:1). The obtained m values were applied to the Median Effect Equation (Equation (2), see Section 3) to determine the doses of each inhibitor required to achieve a specific percentage of rhodesain inhibition [40,44].

The m value reflects the shape and steepness of the dose–response curve, providing insight into the cooperativity of an inhibitor’s effect. A higher m value, as observed for curcumin, indicates a steeper dose–response curve, suggesting that small increases in concentration lead to large increases in enzyme inhibition, which is consistent with highly potent binding. In contrast, the lower m value for **1a** reflects a shallower curve, characteristic of a more gradual dose-dependent effect. The intermediate m value for the combination indicates an integrated response of both inhibitors, producing a dose–response profile that is less steep than curcumin alone but steeper than **1a** alone, consistent with partial cooperativity. To evaluate the interaction between the two inhibitors on rhodesain activity, the Chou and Talalay method was applied. The combination index (CI) was calculated to classify the nature of their interaction as synergistic, additive, or antagonistic. Considering that curcumin is a non-competitive inhibitor [24] and that **1a** acts as a competitive inhibitor of rhodesain, the equation for mutually non-exclusive drugs, which act independently (Equation (3), Section 3), was used to calculate CI. A CI value greater than 1 indicates antagonism, a value equal to 1 indicates an additive effect, and a value less than 1 indicates synergism [45].

**Figure 6:**
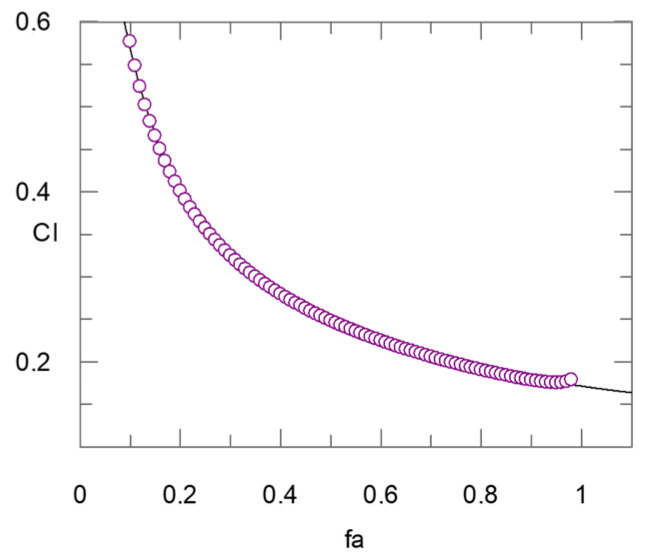
Graphical presentation of the combination index (CI) *versus* affected fraction (f_a_), i.e., the reduction effect of rhodesain activity obtained from a mixture of curcumin-**1a** (molar ratio 0.37:1).

The CI values obtained for the curcumin–**1a** combination were consistently below 1 across the entire range of affected fraction (f_a_ = 0.1–0.90), indicating a robust synergism. Notably, CI values decreased progressively with increasing f_a_, reaching values below 0.3 at f_a_ ≥ 0.3 and falling below 0.2 at f_a_ ≥ 0.8, suggesting a particularly strong synergistic effect at higher levels of enzyme inhibition (Figure 6, Table 3). This trend indicates that while the combination exhibits measurable synergism at low inhibition levels, the effect is greatly amplified as rhodesain inhibition increases, highlighting a dose-dependent enhancement of activity.

Mechanistically, the synergistic effect likely arises from the complementary modes of inhibition: curcumin binds to an allosteric site, altering the enzyme conformation, while **1a** competes with the substrate at the active site. This dual targeting may enhance enzyme inactivation, as curcumin-induced conformational changes could facilitate substrate displacement by **1a**, amplifying the overall inhibitory effect.

Such synergistic interactions suggest that the combination of curcumin and **1a** could achieve effective rhodesain inhibition at lower individual doses, which may reduce potential off-target effects and improve the therapeutic index. These findings provide a strong rationale for further preclinical evaluation of this combination as a strategy to target rhodesain-dependent pathways.

Finally, selectivity assays were carried out (Table 4) by testing inhibitors against human cathepsin L. Fluorometric assays were carried out using Cbz-Phe-Arg-AMC (5 mM) as fluorogenic substrate. A preliminary screening was performed at a fixed inhibitor concentration (100 µM), and only compounds that inhibited at least 70% of enzyme activity were further tested at seven different concentrations to determine the dissociation constants (*K*_i_) of each compound. The results of this investigation clearly pointed out that the combination is more selective than curcumin or chalcone **1a** alone, thus confirming the utility to be used in combination.

### 2.3. Molecular Modeling Studies

**Figure 7:**
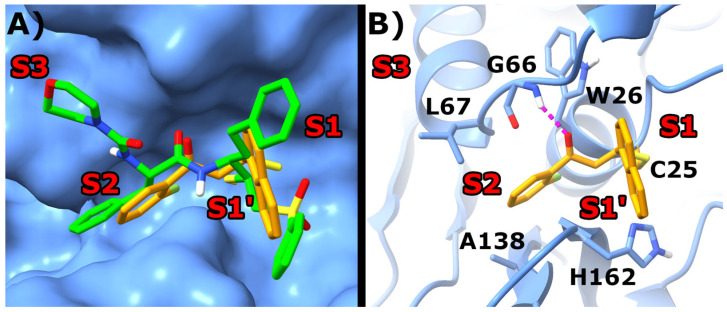
(**A**) Superposition between K11002 (green sticks) and **1a** (orange sticks). Rhodesain is depicted as a blue surface. Enzyme subpockets are labeled in red. (**B**) Predicted binding mode of **1a** (orange sticks) in rhodesain (blue sticks and ribbons) catalytic site. H-bond interactions are depicted as magenta dashed lines. Enzyme subpockets are labeled in red.

Molecular docking simulations were undertaken to establish a theoretical model elucidating the binding modes of the novel chalcone derivatives within the rhodesain binding site. These calculations were based on the X-ray crystal structure of rhodesain bound to the K11002 inhibitor (PDB code: 2P86) [11]. Given that the investigated compounds form covalent adducts with the catalytic C25 residue, we applied a covalent docking protocol utilizing the “flexible side-chain method” implemented in AutoDock4 (AD4) [46]. This technique modifies the interacting amino acid by attaching the ligand directly to its side chain and subsequently treating the resulting complex as flexible throughout the entire docking process. Compound **1a** was chosen for detailed analysis as it exhibited the highest affinity for rhodesain among this series of derivatives. Specifically, the covalent docking of **1a** simulated the 1,4-conjugate addition between the C25 thiol group and the β-carbon of the chalcone’s α,β-unsaturated carbonyl moiety (Figure 7). To further refine the predicted binding geometries, the lowest energy conformation predicted by AD4 was subjected to an additional minimization round. The refined conformation reveals that compound **1a** binds within the enzyme’s active site by closely mirroring the crystallographic pose of the reference inhibitor K11002 (Figure 7A). However, unlike the co-crystal ligand, **1a** is unable to reach the S3 cavity due to a lack of structural extension in that region of the molecule.

On the other hand, the 1-naphthyl moiety (shared by compounds **1a-i**) is placed between the S1 and S1′ subpockets, occupying an electron-rich region defined by residues such as W26 and H162, respectively (Figure 7B). This orientation facilitates the formation of a hydrogen bond by optimally directing the carbonyl group toward the G66 backbone NH, which, in turn, helps anchor the phenyl ring within the S2 cavity. Here, the ligand penetrates deep into the gorge, primarily driven by a variety of hydrophobic contacts with nearby residues lining this specific subpocket, such as L67 and A138. When analyzing the reported structure-activity relationship (SAR) data for compounds **1a-i** as well as **2a-i,** the effect of the phenyl substitution pattern cannot be easily rationalized through the analysis of the static picture obtained from docking studies. In particular, we postulate that these substituents might affect the reactivity of the newly described chalcones as well as the energetic stability of the final ligand-protein covalent adduct. In this view, in the **1** derivatives, **1a** by featuring the *ortho*-F electron-withdrawing substituent should enhance the ligand’s reactivity by enhancing the electrophilic nature of the Michael’s acceptor β-carbon atom while assuring the steric and electrostatic complementarity in the final complex. On the other hand, the bromine analogue **1g** should have a more pronounced effect on the complex stability, while the chlorine ones **1d-f** have a mixed effect that results in lower inhibition potencies.

## 3. Materials and Methods

### 3.1. Chemistry

All reagents and solvents were obtained from commercial suppliers and were used without further purification. Elemental analyses were carried out on a C. Erba Model 1106 Elemental Analyser for C, H and N (Cornaredo, Milan, Italy), and the obtained results are within ±0.4% of the theoretical values. Merck silica gel 60 F254 plates (Merck, Darmstadt, Germany) were used for analytical TLC; flash column chromatography was performed on Merck silica gel (200–400 mesh). ^1^H and ^13^C and NMR spectra were recorded on a Varian 500 MHz spectrometer equipped with a ONE_NMR probe and operating at 499.74 and 125.73 MHz for ^1^H and ^13^C and NMR spectra, respectively. We used the residual signal of the deuterated solvent as an internal standard. Splitting patterns are described as singlet (s), doublet (d), doublet of doublet (dd), triplet (t), quartet (q), multiplet (m), or broad singlet (bs). ^1^H and ^13^C NMR chemical shifts (δ) are expressed in ppm, and coupling constants (*J*) are given in Hz (See the Supplementary Data, Figure S1–S32).

### 3.2. Synthesis of Compounds **1**-**2 a**-**i**

**(*E*)-1-(2-fluorophenyl)-3-(naphtalen-5-yl)prop-2-en-1-one (1a)**

Into a 50 mL flask containing a hydroalcoholic solution (8 mL H_2_O + 6.6 mL EtOH) of sodium hydroxide (64.02 mg, 1.60 mmol), previously placed in an ice bath at a temperature of 0 °C, the 1-naphthaldehyde **3** (43.48 µL, 0.32 mmol) and the 2-fluoroacetophenone **5a** (38.90 µL, 0.32 mmol) were charged. The reaction mixture was then stirred at room temperature and monitored using TLC with petroleum ether/diethyl ether 9:1 as the solvent system. After the reagents disappeared, the solvent was evaporated in vacuo, and then the aqueous phase was extracted with DCM, washed with a saturated NaCl solution, dried over Na_2_SO_4_, filtered and concentrated in vacuo. The residue was purified by silica gel column chromatography using petroleum ether/diethyl ether 9:1 to obtain the pure product **1a** (58 mg, 65%); Consistency: yellow powder; R_f_ = 0.43 (petroleum ether/diethyl ether 9:1). ^1^H NMR (500 MHz, CDCl_3_) = δ: 7.17–7.22 (m, 1H), 7.29 (t, *J* = 7.5 Hz, 1H), 7.47–7.62 (m, 5H), 7.88–7.91 (m, 3H), 7.92 (d, *J* = 8.4 Hz, 1H), 8.24 (d, *J* = 8.4 Hz, 1H), 8.62 (d, *J* = 15.8 Hz, 1H) ppm; ^13^C NMR (125.73 MHz, CDCl_3_) = δ: 116.58 (d, *J* = 23.2 Hz), 123.40, 124.55, 125.34, 125.46, 126.27, 126.98, 128.03, 128.08, 128.77, 130.94, 131.08, 131.77, 132.05, 133.72, 134.03 (d, *J* = 8.8 Hz), 141.62, 161.32 (d, *J* = 253.2 Hz), 188.88 ppm. Elemental analysis: calcd for C_19_H_13_FO: C, 82.59; H, 4.74; found: C 82.21, H 4.99.

**(*E*)-1-(3-fluorophenyl)-3-(naphtalen-5-yl)prop-2-en-1-one (1b)**

Into a 50 mL flask containing a hydroalcoholic solution of sodium hydroxide (64.02 mg, 1.60 mmol), according to the same procedure described for **1a**, 1-naphthaldehyde **3** (43.48 µL, 0.32 mmol) and the 3-fluoroacetophenone **5b** (39.27 µL, 0.32 mmol) were charged a–nd a precipitate was obtained. It was purified by silica gel column chromatography using petroleum ether/diethyl ether 9:1 to obtain the pure product **1b** (51.6 mg, 57%); Consistency: yellow powder; R_f_ = 0.52 (petroleum ether/diethyl ether 9:1). ^1^H NMR (500 MHz, CDCl_3_) = δ: 7.31 (t, *J* = 8.0 Hz, 1H), 7.48–7.58 (m, 4H), 7.58–7.63 (m, 1H), 7.77 (d, *J* = 9.4 Hz, 1H), 7.85–7.88 (m, 1H), 7.88–7.93 (m, 2H), 7.95 (d, *J* = 8.3 Hz, 1H), 8.26 (d, *J* = 8.3 Hz, 1H), 8.70 (d, *J* = 15.4 Hz, 1H) ppm; ^13^C NMR (125.73 MHz, CDCl_3_) = δ: 115.35 (d, *J* = 22.4 Hz), 119.86 (d, *J* = 21.5 Hz), 123.39, 124.03, 124.22, 125.18, 125.42, 126.36, 127.07, 128.79, 130.31 (d, *J* = 7.7 Hz), 131.07, 131.75, 132.10, 133.74, 140.32, 142.45, 162.91 (d, *J* = 248.0 Hz), 188.86 ppm. Elemental analysis: calcd for C_19_H_13_FO: C, 82.59; H, 4.74; found: C 82.88, H 5.06.

**(*E*)-1-(4-fluorophenyl)-3-(naphtalen-5-yl)prop-2-en-1-one (1c)**

Into a 50 mL flask containing a hydroalcoholic solution of sodium hydroxide (64.02 mg, 1.60 mmol), according to the same procedure described for **1a**, 1-naphthaldehyde **3** (43.48 µL, 0.32 mmol) and the 4-fluoroacetophenone **5c** (38.86 µL, 0.32 mmol) were charged and a precipitate was obtained. It was purified by silica gel column chromatography using petroleum ether/diethyl ether 9:1 to obtain the pure product **1c** (54.1 mg, 62%); Consistency: yellow powder; R_f_ = 0.49 (petroleum ether/diethyl ether 9:1). ^1^H NMR (500 MHz, CDCl3) = δ: 7.20 (t, *J* = 8.5 Hz, 2H), 7.50–7.57 (m, 2H), 7.58–7.64 (m, 2H), 7.90 (d, *J* = 7.3 Hz, 2H), 7.94 (d, *J* = 8.3 Hz, 1H), 8.08–8.16 (m, 2H), 8.26 (d, *J* = 8.3 Hz, 1H), 8.68 (d, *J* = 15.4 Hz, 1H) ppm; ^13^C NMR (125.73 MHz, CDCl_3_) = δ: 115.79 (d, *J* = 21.9 Hz), 123.44, 124.17, 125.10, 125.41, 126.34, 127.02, 128.77, 130.91, 131.16 (d, *J* = 9.2 Hz), 131.75, 132.25, 133.73, 134.48, 141.96, 165.65 (d, *J* = 254.6 Hz), 188.57 ppm. Elemental analysis: calcd for C_19_H_13_FO: C, 82.59; H, 4.74; found: C 82.27, H 4.47.

**(*E*)-1-(2-chlorophenyl)-3-(naphtalen-5-yl)prop-2-en-1-one (1d)**

Into a 50 mL flask containing a hydroalcoholic solution of sodium hydroxide (64.02 mg, 1.60 mmol), according to the same procedure described for **1a**, 1-naphthaldehyde **3** (43.48 µL, 0.32 mmol) and the 2-chloroacetophenone **5d** (37.38 µL, 0.32 mmol) were charged and a crude residue was obtained. It was purified by silica gel column chromatography using petroleum ether/diethyl ether 9:1 to obtain the pure product **1d** (60.5 mg, 64%). Consistency: yellow powder; R_f_ = 0.59 (petroleum ether/diethyl ether 9:1). ^1^H NMR (500 MHz, CDCl_3_) = δ: 7.25 (d, *J* = 15.8 Hz, 1H), 7.41 (t, *J* = 7.5 Hz, 1H), 7.45 (t, *J* = 7.9 Hz, 1H), 7.48–7.59 (m, 5H), 7.86 (d, *J* = 7.1 Hz, 1H), 7.88 (d, *J* = 7.8 Hz, 1H), 7.92 (d, *J* = 8.2 Hz, 1H), 8.10 (d, *J* = 8.2 Hz, 1H), 8.37 (d, *J* = 15.8 Hz, 1H) ppm; ^13^C NMR (125.73 MHz, CDCl_3_) = δ: 123.13, 125.41, 125.44, 126.27, 126.92, 127.01, 128.54, 128.78, 129.50, 130.33, 131.10, 131.37, 131.53, 131.55, 131.68, 133.67, 139.17, 142.87, 193.51 ppm. Elemental analysis: calcd for C_19_H_13_ClO: C, 77.95; H, 4.48; found: C 78.22, H 4.74.

**(*E*)-1-(3-chlorophenyl)-3-(naphtalen-5-yl)prop-2-en-1-one (1e)**

Into a 50 mL flask containing a hydroalcoholic solution of sodium hydroxide (64.02 mg, 1.60 mmol), according to the same procedure described for **1a**, 1-naphthaldehyde **3** (43.48 µL, 0.32 mmol) and the 3-chloroacetophenone **5e** (41.56 µL, 0.32 mmol) were charged and a precipitate was obtained. It was purified by silica gel column chromatography using petroleum ether/diethyl ether 9:1 to obtain the pure product **1e** (57.7 mg, 62%); Consistency: yellow powder; R_f_ = 0.50 (petroleum ether/diethyl ether 9:1). ^1^H NMR (500 MHz, CDCl_3_) = δ: 7.47 (t, *J* = 7.6 Hz, 1H), 7.52–7.63 (m, 5H), 7.91 (t, *J* = 7.6 Hz, 2H), 7.95 (d, *J* = 8.4 Hz, 2H), 8.05 (s, 1H), 8.25 (d, *J* = 8.4 Hz, 1H), 8.70 (d, *J* = 15.4 Hz, 1H) ppm; ^13^C NMR (125.73 MHz, CDCl_3_) = δ: 123.40, 123.93, 125.23, 125.44, 126.38, 126.63, 127.11, 128.67, 128.82, 130.01, 131.14, 131.76, 132.05, 132.80, 133.75,135.00, 139.76, 142.56, 188.87 ppm. Elemental analysis: calcd for C_19_H_13_ClO: C, 77.95; H, 4.48; found: C 77.83, H 4.69.

**(*E*)-1-(4-chlorophenyl)-3-(naphtalen-5-yl)prop-2-en-1-one (1f)**

Into a 50 mL flask containing a hydroalcoholic solution of sodium hydroxide (64.02 mg, 1.60 mmol), according to the same procedure described for **1a**, 1-naphthaldehyde **3** (43.48 µL, 0.32 mmol) and the 4-chloroacetophenone **5f** (41.52 µL, 0.32 mmol) were charged and a precipitate was obtained. It was purified by silica gel column chromatography using petroleum ether/diethyl ether 9:1 to get the pure product **1f** (52 mg, 55%); Consistency: yellow powder; R_f_ = 0.57 (petroleum ether/diethyl ether 9:1). ^1^H NMR (500 MHz, CDCl_3_) = δ: 7.45–7.64 (m, 6H), 7.90 (d, *J* = 6.9 Hz, 2H), 7.93 (d, *J* = 8.2 Hz, 1H), 8.02 (d, *J* = 6.9 Hz, 2H), 8.24 (d, *J* = 8.2 Hz, 1H), 8.68 (d, *J* = 15.4 Hz, 1H) ppm; ^13^C NMR (125.73 MHz, CDCl_3_) = δ: 123.42, 124.07, 125.15, 125.43, 126.37, 127.07, 128.80, 129.00, 129.99, 131.02, 131.76, 132.18, 133.75, 136.48, 139.32, 142.25, 188.95 ppm. Elemental analysis: calcd for C_19_H_13_ClO: C, 77.95; H, 4.48; found: C 77.59, H 4.10.

**(*E*)-1-(2-bromophenyl)-3-(naphtalen-5-yl)prop-2-en-1-one (1g)**

Into a 50 mL flask containing a hydroalcoholic solution of sodium hydroxide (64.02 mg, 1.60 mmol), according to the same procedure described for **1a**, 1-naphthaldehyde **3** (43.48 µL, 0.32 mmol) and the 2-bromoacetophenone **5g** (43.17 µL, 0.32 mmol) were charged and a crude residue was obtained. It was purified by silica gel column chromatography using petroleum ether/diethyl ether 85:15 to obtain the pure product **1g** (75.6 mg, 70%); Consistency: yellow oil; R_f_ = 0.40 (petroleum ether/diethyl ether 85:15). ^1^H NMR (500 MHz, CDCl_3_) = δ: 7.22 (d, *J* = 15.8 Hz, 1H), 7.37 (t, *J* = 6.4 Hz, 1H), 7.45 (t, *J* = 7.0 Hz, 1H), 7.49–7.58 (m, 4H), 7.69 (d, *J* = 8.0 Hz, 1H), 7.87 (t, *J* = 8.2 Hz, 2H), 7.92 (d, *J* = 8.2 Hz, 1H), 8.08 (d, *J* = 8.2 Hz, 1H), 8.34 (d, *J* = 15.8 Hz, 1H) ppm; ^13^C NMR (125.73 MHz, CDCl_3_) = δ: 119.67, 123.23, 125.56, 125.59, 126.42, 127.17, 127.58, 128.49, 128.76, 128.93, 129.45, 131.29, 131.64, 131.76, 133.60, 133.78, 141.33, 143.36, 194.55 ppm. Elemental analysis: calcd for C_19_H_13_BrO: C, 67.67; H, 3.89; found: C 67.49, H 4.08.

**(*E*)-1-(3-bromophenyl)-3-(naphtalen-5-yl)prop-2-en-1-one (1h)**

Into a 50 mL flask containing a hydroalcoholic solution of sodium hydroxide (64.02 mg, 1.60 mmol), according to the same procedure described for **1a**, 1-naphthaldehyde **3** (43.48 µL, 0.32 mmol) and the 3-bromoacetophenone **5h** (42.34 µL, 0.32 mmol) were charged and a crude residue was obtained. It was purified by silica gel column chromatography using petroleum ether/diethyl ether 9:1 to obtain the pure product **1h** (84 mg, 78%). Consistency: yellow powder; R_f_ = 0.52 (petroleum ether/diethyl ether 9:1). ^1^H NMR (500 MHz, CDCl_3_) = δ: 7.40 (t, *J* = 7.8 Hz, 1H), 7.51–7.62 (m, 4H), 7.72 (d, *J* = 7.8 Hz, 1H), 7.90 (t, *J* = 6.9 Hz, 2H), 7.94 (d, *J* = 8.3 Hz, 1H), 7.99 (d, *J* = 7.8 Hz, 1H), 8.20 (s, 1H), 8.25 (d, *J* = 8.3 Hz, 1H), 8.69 (d, *J* = 15.4 Hz, 1H) ppm; ^13^C NMR (125.73 MHz, CDCl_3_) = δ: 123.02, 123.38, 123.88, 125.22, 125.43, 126.36, 127.05, 127.09, 128.81, 130.24, 131.12, 131.58, 131.75, 132.02, 133.73, 135.68, 139.96, 142.53, 188.72 ppm. Elemental analysis: calcd for C_19_H_13_BrO: C, 67.67; H, 3.89; found: C 67.28, H 3.52.

**(*E*)-1-(4-bromophenyl)-3-(naphtalen-5-yl)prop-2-en-1-one (1i)**

Into a 50 mL flask containing a hydroalcoholic solution of sodium hydroxide (64.02 mg, 1.60 mmol), according to the same procedure described for **1a**, 1-naphthaldehyde **3** (43.48 µL, 0.32 mmol) and the 4-bromoacetophenone **5i** (63.65 mg, 0.32 mmol) were charged and a crude residue was obtained. It was purified by silica gel column chromatography using petroleum ether/diethyl ether 9:1 to obtain the pure product **1i** (64 mg, 60%). Consistency: yellow powder; R_f_ = 0.48 (petroleum ether/diethyl ether 9:1). The NMR data (^1^H and ^13^C NMR) are in agreement with those reported in the literature [47]. Elemental analysis: calcd for C_19_H_13_BrO: C, 67.67; H, 3.89; found: C 67.58, H 3.95.

**(*E*)-1-(2-fluorophenyl)-3-(naphtalen-6-yl)prop-2-en-1-one (2a)**

Into a 50 mL flask containing a hydroalcoholic solution of sodium hydroxide (64.02 mg, 1.60 mmol), according to the same procedure described for **1a**, 2-naphthaldehyde **4** (50 mg, 0.32 mmol) and the 2-fluoroacetophenone **5a** (38.90 µL, 0.32 mmol) were charged and a precipitate was obtained. It was purified by silica gel column chromatography using petroleum ether/diethyl ether 9:1 to obtain the pure product **2a** (52.5 mg, 59%); Consistency: pale yellow powder; R_f_ = 0.42 (petroleum ether/diethyl ether 9:1). ^1^H NMR (500 MHz, CDCl_3_) = δ: 7.07–7.11 (m, 1H), 7.17 (t, *J* = 6.7 Hz, 1H), 7.39–7.46 (m, 4H), 7.66 (d, *J* = 7.3 Hz, 1H), 7.73–7.78 (m, 4H), 7.81 (d, *J* = 15.8 Hz, 1H), 7.90 (s, 1H) ppm; ^13^C NMR (125.73 MHz, CDCl_3_) = δ: 116.55 (d, *J* = 23.1 Hz), 123.72, 124.54, 125.71, 125.76, 126.75, 127.43, 127.80, 128.66, 128.73, 130.88, 130.98, 133.21, 133.31, 133.88 (d, *J* = 8.8 Hz), 134.45, 144.97, 161.22 (d, *J* = 253.1 Hz), 189.01 ppm. Elemental analysis: calcd for C_19_H_13_FO: C, 82.59; H, 4.74; found: C 82.90, H 5.00.

**(*E*)-1-(3-fluorophenyl)-3-(naphtalen-6-yl)prop-2-en-1-one (2b)**

Into a 50 mL flask containing a hydroalcoholic solution of sodium hydroxide (64.02 mg, 1.60 mmol), according to the same procedure described for **1a**, 2-naphthaldehyde **4** (50 mg, 0.32 mmol) and the 3-fluoroacetophenone **5b** (39.27 µL, 0.32 mmol) were charged. The compound **2b** precipitated (55 mg, 62%), so it was filtered using a Büchner funnel under vacuum and washed with H_2_O without any further purification procedures. Consistency: yellow powder; R_f_ = 0.38 (petroleum ether/diethyl ether 9:1). ^1^H NMR (500 MHz, CDCl_3_) = δ: 7.30 (t, *J* = 8.2 Hz, 1H), 7.49–7.56 (m, 3H), 7.59 (d, *J* = 15.6 Hz, 1H), 7.74 (d, *J* = 9.5 Hz, 1H), 7.80 (d, *J* = 8.6 Hz, 1H), 7.83–7.93 (m, 4H), 8.00 (d, *J* = 15.6 Hz, 1H), 8.04 (s, 1H) ppm; ^13^C NMR (125.73 MHz, CDCl_3_) = δ: 115.30 (d, *J* = 22.4 Hz), 119.75 (d, *J* = 21.4 Hz), 121.60, 123.58, 124.18, 126.83, 127.51, 127.81, 128.68, 128.80, 130.27 (d, *J* = 7.7 Hz), 130.90, 132.13, 133.33, 134.48, 140.43, 145.65, 162.89 (d, *J* = 248.0 Hz), 189.07 ppm. Elemental analysis: calcd for C_19_H_13_FO: C, 82.59; H, 4.74; found: C 82.33, H 4.38.

**(*E*)-1-(4-fluorophenyl)-3-(naphtalen-6-yl)prop-2-en-1-one (2c)**

Into a 50 mL flask containing a hydroalcoholic solution of sodium hydroxide (64.02 mg, 1.60 mmol), according to the same procedure described for **1a**, 2-naphthaldehyde **4** (50 mg, 0.32 mmol) and the 4-fluoroacetophenone **5c** (38.86 µL, 0.32 mmol) were charged and a precipitate was obtained. It was purified by silica gel column chromatography using petroleum ether/diethyl ether 9:1 to get the pure product **2c** (47.5 mg, 54%); Consistency: pale yellow powder; R_f_ = 0.43 (petroleum ether/diethyl ether 9:1). ^1^H NMR (500 MHz, CDCl_3_) = δ: 7.19 (t, *J* = 8.6 Hz, 2H), 7.46–7.57 (m, 2H), 7.61 (d, *J* = 15.6 Hz, 1H), 7.78 (d, *J* = 8.6 Hz, 1H), 7.82–7.90 (m, 3H), 7.98 (d, *J* = 15.5 Hz, 1H), 8.03 (s, 1H), 8.06–8.13 (m, 2H) ppm; ^13^C NMR (125.73 MHz, CDCl_3_) = δ: 115.74 (d, *J* = 21.8 Hz), 121.65, 123.60, 126.80, 127.44, 127.80, 128.65, 128.75, 130.73, 131.09 (d, *J* = 9.2 Hz), 132.24, 133.34, 134.41, 134.58, 145.10, 165.59 (d, *J* = 254.4 Hz), 188.74 ppm. Elemental analysis: calcd for C_19_H_13_FO: C, 82.59; H, 4.74; found: C 82.91, H 4.47.

**(*E*)-1-(2-chlorophenyl)-3-(naphtalen-6-yl)prop-2-en-1-one (2d)**

Into a 50 mL flask containing a hydroalcoholic solution of sodium hydroxide (64.02 mg, 1.60 mmol), according to the same procedure described for **1a**, 2-naphthaldehyde **4** (50 mg, 0.32 mmol) and the 2-chloroacetophenone **5d** (37.38 µL, 0.32 mmol) were charged. The compound **2d** precipitated (53.6 mg, 57%), and it was filtered using a Büchner funnel under vacuum and washed with H_2_O without any further purification procedures. Consistency: white powder; R_f_ = 0.38 (petroleum ether/diethyl ether 9:1). ^1^H NMR (500 MHz, CDCl_3_) = δ: 7.29 (s, 1H), 7.42 (t, *J* = 7.4 Hz, 1H), 7.48 (t, *J* = 6.6 Hz, 1H), 7.50–7.61 (m, 4H), 7.66 (d, *J* = 16.1 Hz, 1H), 7.76 (d, *J* = 8.5 Hz, 1H), 7.88 (t, *J* = 7.1 Hz, 3H), 7.99 (s, 1H) ppm; ^13^C NMR (125.73 MHz, CDCl_3_) = δ: 123.58, 126.43, 126.81, 126.85, 127.56, 127.81, 128.65, 128.81, 129.35, 130.30, 130.92, 131.32, 131.36, 131.93, 133.25, 134.49, 139.18, 146.40, 193.82 ppm. Elemental analysis: calcd for C_19_H_13_ClO: C, 77.95; H, 4.48; found: C 77.58, H 4.72.

**(*E*)-1-(3-chlorophenyl)-3-(naphtalen-6-yl)prop-2-en-1-one (2e)**

Into a 50 mL flask containing a hydroalcoholic solution of sodium hydroxide (64.02 mg, 1.60 mmol), according to the same procedure described for **1a**, 2-naphthaldehyde **4** (50 mg, 0.32 mmol) and the 3-chloroacetophenone **5e** (41.56 µL, 0.32 mmol) were charged. The compound **2e** precipitated (60 mg, 64%), so it was filtered using a Büchner funnel under vacuum and washed with H_2_O without any further purification procedures. Consistency: yellow powder; R_f_ = 0.40 (petroleum ether/diethyl ether 9:1). ^1^H NMR (500 MHz, CDCl_3_) = δ: 7.47 (t, *J* = 6.9 Hz, 1H), 7.51–7.61 (m, 4H), 7.80 (d, *J* = 6.5 Hz, 1H), 7.83–7.91 (m, 3H), 7.93 (d, *J* = 7.7 Hz, 1H), 7.99 (d, *J* = 15.7 Hz, 1H), 8.03 (s, 1H), 8.05 (s, 1H) ppm; ^13^C NMR (125.73 MHz, CDCl_3_) = δ: 121.69, 123.76, 126.72, 127.00, 127.70, 127.98, 128.74, 128.85, 128.97, 130.12, 131.11, 132.27, 132.83, 133.49, 134.66, 135.11, 140.04, 145.93, 189.24 ppm. Elemental analysis: calcd for C_19_H_13_ClO: C, 77.95; H, 4.48; found: C 77.72, H 4.19.

**(*E*)-1-(4-chlorophenyl)-3-(naphtalen-6-yl)prop-2-en-1-one (2f)**

Into a 50 mL flask containing a hydroalcoholic solution of sodium hydroxide (64.02 mg, 1.60 mmol), according to the same procedure described for **1a**, 2-naphthaldehyde **4** (50 mg, 0.32 mmol) and the 4-chloroacetophenone **5f** (41.52 µL, 0.32 mmol) were charged. The compound **2f** precipitated (50.6 mg, 54%), so it was filtered using a Büchner funnel under vacuum and washed with H_2_O without any further purification procedures. Consistency: pale yellow powder; R_f_ = 0.47 (petroleum ether/diethyl ether 9:1). ^1^H NMR (500 MHz, CDCl_3_) = δ: 7.50 (d, *J* = 8.2 Hz, 2H), 7.52–7.56 (m, 2H), 7.59 (d, *J* = 15.0 Hz, 1H), 7.79 (d, *J* = 8.6 Hz, 1H), 7.83–7.92 (m, 3H), 7.97 (d, *J* = 15.0 Hz, 1H), 8.00 (s, 1H), 8.03 (d, *J* = 12.1 Hz, 2H) ppm; ^13^C NMR (125.73 MHz, CDCl_3_) = δ: 121.57, 123.58, 126.82, 127.49, 127.80, 128.66, 128.78, 128.94, 129.91, 130.83, 132.18, 133.33, 134.45, 136.57, 139.20, 145.42, 189.11 ppm. Elemental analysis: calcd for C_19_H_13_ClO: C, 77.95; H, 4.48; found: C 77.78, H 4.69.

**(*E*)-1-(2-bromophenyl)-3-(naphtalen-6-yl)prop-2-en-1-one (2g)**

Into a 50 mL flask containing a hydroalcoholic solution of sodium hydroxide (64.02 mg, 1.60 mmol), according to the same procedure described for **1a**, 2-naphthaldehyde **4** (50 mg, 0.32 mmol) and the 2-bromoacetophenone **5g** (43.17 µL, 0.32 mmol) were charged and a precipitate was obtained. It was purified by silica gel column chromatography using petroleum ether/diethyl ether 9:1 to obtain the pure product **2g** (93.8 mg, 87%); Consistency: pale yellow powder; R_f_ = 0.35 (petroleum ether/diethyl ether 9:1). ^1^H NMR (500 MHz, CDCl_3_) = δ: 7.22 (d, *J* = 16.1 Hz, 1H), 7.35 (t, *J* = 6.8 Hz, 1H), 7.43 (t, *J* = 7.4 Hz, 1H), 7.45–7.55 (m, 3H), 7.60 (d, *J* = 16.1 Hz, 1H), 7.67 (d, *J* = 8.0 Hz, 1H), 7.71 (d, *J* = 8.6 Hz, 1H), 7.81–7.87 (m, 3H), 7.93 (s, 1H) ppm; ^13^C NMR (125.73 MHz, CDCl_3_) = δ: 119.62, 123.65, 126.35, 126.92, 127.47, 127.68, 127.91, 128.75, 128.92, 129.28, 131.04, 131.45, 131.99, 133.32, 133.54, 134.59, 141.30, 146.82, 194.75 ppm. Elemental analysis: calcd for C_19_H_13_BrO: C, 67.67; H, 3.89; found: C 67.98, H 4.12.

**(*E*)-1-(3-bromophenyl)-3-(naphtalen-6-yl)prop-2-en-1-one (2h)**

Into a 50 mL flask containing a hydroalcoholic solution of sodium hydroxide (64.02 mg, 1.60 mmol), according to the same procedure described for **1a**, 2-naphthaldehyde **4** (50 mg, 0.32 mmol) and the 3-bromoacetophenone **5h** (42.34 µL, 0.32 mmol) were charged. The compound **2h** precipitated (82 mg, 76%), so it was filtered using a Büchner funnel under vacuum and washed with H_2_O without any further purification procedures. Consistency: pale yellow powder; R_f_ = 0.47 (petroleum ether/diethyl ether 9:1). ^1^H NMR (500 MHz, CDCl_3_) = δ: 7.41 (t, *J* = 7.8 Hz, 1H), 7.51–7.56 (m, 2H), 7.57 (d, *J* = 15.4 Hz, 1H), 7.73 (d, *J* = 8.0 Hz, 1H), 7.80 (d, *J* = 8.6 Hz, 1H), 7.84–7.92 (m, 3H), 7.96–8.02 (m, 2H), 8.05 (s, 1H), 8.18 (s, 1H) ppm; ^13^C NMR (125.73 MHz, CDCl_3_) = δ: 121.47, 122.98, 123.60, 126.84, 127.00, 127.54, 127.81, 128.69, 128.80, 130.21, 130.96, 131.50, 132.10, 133.32, 134.50, 135.58, 140.08, 145.78, 188.95 ppm. Elemental analysis: calcd for C_19_H_13_BrO: C, 67.67; H, 3.89; found: C 67.35, H 3.56.

**(*E*)-1-(4-bromophenyl)-3-(naphtalen-6-yl)prop-2-en-1-one (2i)**

Into a 50 mL flask containing a hydroalcoholic solution of sodium hydroxide (64.02 mg, 1.60 mmol), according to the same procedure described for **1a**, 2-naphthaldehyde **4** (50 mg, 0.32 mmol) and the 4-bromoacetophenone **5i** (63.65 mg, 0.32 mmol) were charged. The compound **2i** precipitated (85 mg, 80%), so it was filtered using a Büchner funnel under vacuum and washed with H_2_O without any further purification procedures. Consistency: pale yellow powder; R_f_ = 0.42 (petroleum ether/diethyl ether 9:1). The NMR data (^1^H and ^13^C NMR) are in agreement with those reported in the literature [47]. Elemental analysis: calcd for C_19_H_13_BrO: C, 67.67; H, 3.89; found: C 67.54, H 4.12.

### 3.3. Rhodesain Inhibition Assays

Initial screening of rhodesain inhibition was conducted using fixed concentrations of inhibitors, 100 µM and 10 µM, with an equivalent volume of DMSO used as a negative control, and an equivalent volume of E-64 as a positive control. The enzyme was produced recombinantly according to the procedure described in [48]. Hydrolysis of the substrate Cbz-Phe-Arg-AMC (10 µM) was monitored in real time over 10 min at room temperature by measuring the release of the fluorescent product. The *K*_m_ values used to correct *K*_iapp_ values were 0.9 μM. The assay buffer contains: 50 mM sodium acetate, pH = 5.5, 5 mM EDTA, 200 mM NaCl, and 0.005% Brij, a nonionic polyoxyethylene surfactant, to avoid aggregation and wrong positive results. Enzyme buffer contains 5 mM Dithiothreitol (DTT) rather than Brij. Compounds (**1-2 a-i**) showing at least 70% inhibition at 100 μM and curcumin were tested in independent experiments in duplicate in 96-well plates (BRAND^®^, Wertheim, Germany) in a total volume of 200 µL at seven different concentrations. The concentrations used for the combination study are: (i) for curcumin 1 µM, 5 μM, 10 μM, 20 μM, 40 μM, 60 μM and 80 μM; (ii) for **1a** 5 µM, 10 μM, 20 μM, 40 μM, 60 μM, 80 μM and 100 μM.

AMC fluorescence generated during substrate hydrolysis was measured at room temperature using an Infinite 200 PRO microplate reader (Tecan, Männedorf, Switzerland) with excitation and emission filters of 380 nm and 460 nm, respectively. IC_50_ and *K*_i_ values, reported as mean ± SD, were calculated by fitting the progress curves to the four-parameter dose–response model using the software GraphPad Prism 5.0.3 (GraphPad software Inc., San Diego, CA, USA).

(1) $$y=\frac{y_{max}-y_{min}}{1+{\left(\frac{\left[I\right]}{{IC}_{50}}\right)}^{S}}+y_{min}$$

where, y [∆F/min] denotes the substrate hydrolysis rate, y_max_ represents the maximum response observed in the absence of inhibitor ([I] = 0 µM), y_min_ corresponds to the minimum response measured at the highest inhibitor concentration, and s is the Hill coefficient.

### 3.4. Enzyme Assays Towards hCatL

Similarly to rhodesain, the activity towards hCatL was evaluated as reported in the literature, by using Cbz-Phe-Arg-AMC as substrate (5 μM for cathepsin L) [49]. The biological evaluation towards hCatL was performed using the same buffers, reader, fluorogenic substrate, and positive and negative controls as described above for rhodesain, and the preliminary screening was performed at 100 μM. In this case, too, each independent assay was performed twice and in duplicate. The *K*_m_ values used to correct *K*_iapp_ values were 6.5 μM.

### 3.5. Calculation of the Combination Index

The Combination Index for curcumin with **1a** was determined using six data points: 1/128 × IC_50 F1+F2_, 1/64 × IC_50 F1+F2_, 1/4 × IC_50 F1+F2_, 1/2 × IC_50 F1+F2_, IC_50 F1+F2_, and 2 × IC_50 F1+F2_, where F_1_ = curcumin while F_2_ = **1a** (Table 2). After determining the IC_50_ value of the combination *plus* the standard deviation, the Median Effect Plot was obtained from the dose–response curves. The Median Effect Plot is obtained by plotting log(f_a_/f_u_) *versus* log(D) on the x-axis, to calculate the “m” value, which corresponds to the Hill coefficient and describes the sigmoidal nature (S-shaped curve) of the dose–response graph. More in detail, f_a_ corresponds to the affected fraction, the inhibited fraction of the enzyme, f_u_ corresponds to the unaffected fraction, the uninhibited fraction of the enzyme; the amount given by f_a_ and f_u_ is equal to 1.

After calculating the three distinct values of m using Grafit software (version 5.0; Erithacus Software Limited, East Grinstead, West Sussex, UK), the corresponding single doses required to achieve the defined levels of enzyme inhibition were determined using the Median Effect Equation [44,46].

D = IC_50_ [f_a_/f_u_]^1/m^ (2)

The CI was calculated for mutually non-exclusive drugs, which act independently, using the Chou and Talalay method, with the following equation:

CI = [(D)_1_/(IC_50_)_1_] + [(D)_2_/(IC_50_)_2_] + [(D)_1_(D)_2_]/[(IC_50_)_1_(IC_50_)_2_] (3)

(IC_50_)_1_ and (IC_50_)_2_ represent the concentrations of curcumin and **1a** required to achieve 50% inhibition of rhodesain, respectively, whereas D_1_ and D_2_ denote the doses of curcumin and **1a** producing a defined percentage of inhibition, calculated using the Median Effect Equation as previously described. The IC_50_ values were determined from dose–response curves, while D_1_ and D_2_ were derived from the Median Effect Equation. The Combination Index (CI) for rhodesain inhibition ranging from 10% to 100% was calculated using Grafit software.

### 3.6. Statistical Analyses

Data were statistically analyzed using one-way ANOVA followed by Dunnett’s multiple comparison test, with *p*-values < 0.05 considered statistically significant. Comparisons were made with respect to the percentage of rhodesain inhibition by curcumin, **1a**, as well as the combinations curcumin + **1a**. Analyses were conducted using GraphPad Prism 5.0.3, and results are presented as the mean ± standard deviation (SD).

### 3.7. Docking Studies

Molecular docking calculations were conducted using AutoDock4 (v 4.2.6) [50]. To model the formation of the covalent adduct between the ligand and the protein, the “flexible side chain method” was used [46]. During the preparation phase, the ligand was modeled within the Maestro suite to incorporate two additional atoms (a sulfur and a carbon atom) to facilitate the alignement with the C25 residue of the protein. The X-ray crystal structure of rhodesain (PDB code: 2P86) underwent preliminary adjustments for docking purposes by using the integrated into the Schrödinger suite [51]. The subsequent alignment of the ligand with the reactive cysteine was performed using scripts provided on the AutoDock website. Protein grid maps were generated with AutoGrid4, employing ligand atom types as probes. The dimensions of the enzyme grid box were set at 60 Å × 60 Å × 60 Å with a 0.375 Å spacing and centered on the coordinates of the native co-crystallized ligand. Docking simulations were executed using the Lamarckian genetic algorithm (LGA), allowing flexibility for the modified cysteine-ligand moiety. A total of 100 LGA runs were performed, with all other parameters maintained at their default settings. The resulting conformations were clustered based on a root mean square deviation (RMSD) criterion, with solutions differing by less than 2.0 Å being considered part of the same cluster. The clusters were ranked according to their lowest predicted free energy of binding. Finally, the predicted binding poses were refined through an energy minimization protocol consisting of 5000 steps of the steepest descent algorithm, followed by 5000 steps using the conjugate gradient method. Structural visualization and analysis were carried out using UCSF Chimera X version software 1.19 [52].

## 4. Conclusions

In conclusion, in our paper, we carried out a structure–activity relationship analysis of the synthesized chalcone derivatives **1-2 a-i**. This study highlights the critical influence of both scaffold orientation and halogen substitution on rhodesain inhibition. Among all compounds, **1a**, bearing a 1-naphthyl scaffold with an *ortho*-fluoro substituent, emerged as the most potent inhibitor (*K*_i_ = 2.00 ± 0.19 µM), establishing an optimal orientation and electronic environment for active site binding. In the 1-naphthyl series, *ortho*- and *meta*-fluoro substitutions were generally favorable, whereas *para*-fluoro and larger halogen substituents reduced potency, emphasizing the importance of steric and electronic factors. The 2-naphthyl series displayed an enhanced activity for certain *meta*- and *para*-chloro and bromo derivatives compared with their 1-naphthyl analogues, indicating that the 2-naphthyl orientation can better accommodate specific substituents in the binding pocket. Nevertheless, none of the 2-naphthyl derivatives surpassed the activity of **1a**. These results collectively demonstrate that both naphthyl positioning and halogen placement are key determinants of rhodesain inhibitory activity and identify **1a** as the benchmark compound for future optimization efforts within this series. Furthermore, covalent molecular docking studies supported these SAR observations by illustrating the binding mode of **1a**. The in silico model showed a 1,4-conjugate addition with the catalytic Cys25, with the 1-naphthyl moiety occupying the S1/S1′ region and the phenyl ring anchored in the S2 cavity via a hydrogen bond with Gly66. Beyond this foundational binding model, the superior potency of **1a** can be chemically rationalized by the presence of the electron-withdrawing *ortho*-fluoro substituent, which likely enhances the Michael acceptor’s electrophilicity and the overall stability of the covalent adduct.

In addition, in this study, the inhibitory effects of curcumin and compound **1a** against rhodesain were evaluated individually and in combination using the Chou–Talalay method. The slope values “m” derived from Median Effect Plots were m_1_ = 3.9427 for curcumin, m_2_ = 1.0620 for **1a**, and m_1,2_ = 2.9247 for the curcumin–**1a** combination at a molar ratio of 0.37:1. These m values reflect the steepness of the dose–response curves, with curcumin showing a highly cooperative response, **1a** a gradual inhibition profile, and the combination an intermediate behavior indicative of partial cooperativity. Application of the Median Effect Equation allowed calculation of the doses required for specific fractions of rhodesain inhibition and provided the basis for Combination Index (CI) analysis. The CI values were consistently below 1 across the entire range of fractional inhibition (f_a_ = 0.1–0.9), with values decreasing below 0.3 at f_a_ ≥ 0.3 and below 0.2 at f_a_ ≥ 0.8, demonstrating strong synergistic interactions, particularly at higher levels of enzyme inhibition. Mechanistically, this synergy is likely due to the complementary modes of action of the two inhibitors: curcumin binds allosterically, altering the enzyme conformation, while **1a** competes at the active site, resulting in an enhanced enzyme inactivation. Collectively, these results indicate that the combination curcumin–**1a** inhibits rhodesain in a potent synergistic mode, achieving high efficacy at lower individual doses, which may reduce potential off-target effects and improve the therapeutic index as demonstrated by the improved selectivity of the combination with respect to **1a** or curcumin used alone.

This study provides a quantitative foundation for further preclinical evaluation of this combination as a promising strategy for rhodesain-targeted interventions.

## Figures and Tables

**Table 1:** *K*_i_ values of compounds **1-2 a-i** against rhodesain.

Comp.	R	*K*_i_ (µM) or % Inhibition at 100 µM
**1a**	2-F	2.00 ± 0.19
**1b**	3-F	2.61 ± 0.02
**1c**	4-F	58%
**1d**	2-Cl	35%
**1e**	3-Cl	50%
**1f**	4-Cl	44%
**1g**	2-Br	7.16 ± 1.20
**1h**	3-Br	60%
**1i**	4-Br	40%
**2a**	2-F	56%
**2b**	3-F	2.52 ± 1.82
**2c**	4-F	56%
**2d**	2-Cl	58%
**2e**	3-Cl	2.93 ± 0.55
**2f**	4-Cl	2.54 ± 0.31
**2g**	2-Br	52%
**2h**	3-Br	2.95 ± 1.11
**2i**	4-Br	45%

**Table 2:** Six selected doses for the combination assay of curcumin and **1a**.

	1/128 × IC_50_	1/64 × IC_50_	1/4 × IC_50_	1/2 × IC_50_	IC_50_	2 × IC_50_
**Curcumin**	0.20 μM	0.40 μM	6.44 μM	12.88 μM	25.77 μM	51.54 μM
**1a**	0.54 μM	1.08 μM	17.39 μM	34.77 μM	69.55 μM	139.1 μM
**Curcumin +** **1a**	0.20 + 0.54 μM	0.40 + 1.08 μM	6.44 + 17.39 μM	12.88 + 34.77 μM	25.77 + 69.55 μM	51.54 + 139.1 μM

**Table 3:** Combination Index (CI) values of curcumin-**1a** in combination for the reduction in rhodesain activity.

Affected Fraction (f_a_)	% Rhodesain Inhibition	CI	Diagnosis CI
0.1	10%	0.5764	Synergism
0.2	20%	0.4006	Synergism
0.3	30%	0.3247	Synergism
0.4	40%	0.2794	Strong synergism
0.5	50%	0.2481	Strong synergism
0.6	60%	0.2245	Strong synergism
0.7	70%	0.2057	Strong synergism
0.8	80%	0.1902	Strong synergism
0.9	90%	0.1781	Strong synergism

**Table 4:** Biological activity against human cathepsin L.

Compounds	*K*_i_ mM
**1a**	20.0 ± 0.54
Curcumin	2.80 ± 0.16
Curcumin + **1a** (0.37:1)	41.03 ± 2.36

## Data Availability

The original contributions presented in the study are included in the article, further inquiries can be directed to the corresponding author.

## Supplementary Materials

The following supporting information can be downloaded at: https://www.mdpi.com/article/10.3390/ijms27073320/s1.

## Abbreviations

The following abbreviations are used in this manuscript:

CI	Combination Index
NTDs	Neglected tropical diseases
WHO	World Health Organisation
HAT	Human African trypanosomiasis
BBB	Blood–brain barrier
NECT	Nifurtimox-eflornithine combination
EMA	European Medicines Agency
Cbz	Benzyloxycarbonyl
AMC	Aminomethylcumarin
