# Supplementary material for: Synthesis of New Asymmetrical Chalcones and Evaluation of Their Use in Combination with Curcumin Against Rhodesain of T. brucei rhodesiense
Source: Int J Mol Sci. 2026 Apr 7;27(7):3320. doi: 10.3390/ijms27073320 (PMC13072751; doi:10.3390/ijms27073320)
Supplement: Supplementary file 1 [file ijms-27-03320-s001.zip › ijms-4193402-supplementary.pdf]

Supporting information

Synthesis of new asymmetrical chalcones and evaluation of their use in combination with curcumin against rhodesain of *T. brucei rhodesiense*

Carla Di Chio [1,†], Josè Starvaggi [1,†], Benito Natale[2], Santo Previti [1], Fabiola De Luca [1], Sandro Cosconati[2], Tanja Schirmeister [3], Maria Zappalà [1] and Roberta Ettari [1,*]

[1] Department of Chemical, Biological, Pharmaceutical and Environmental Sciences, University of Messina, Viale Ferdinando Stagno d'Alcontres 31, 98166 Messina, Italy;

[2] DiSTABiF, University of Campania Luigi Vanvitelli, Via Vivaldi 43, 81100 Caserta, Italy

[3] Institute of Pharmacy and Biochemistry, University of Mainz, Staudingerweg 5, 55128 Mainz, Germany.

* Correspondence: rettari@unime.it (R.E.); Tel.: Tel.: +39-090-676-6554

† These authors equally contributed to this work.

Figure captions

Figure S1. ^{1}H NMR spectrum of compound **1a.**

Figure S2. ^{13}C NMR spectrum of compound **1a**.

Figure S3. ^{1}H NMR spectrum of compound **1b.**

Figure S4. ^{13}C NMR spectrum of compound **1b**.

Figure S5. ^{1}H NMR spectrum of compound **1c.**

Figure S6. ^{13}C NMR spectrum of compound **1c**.

Figure S7. ^{1}H NMR spectrum of compound **1d.**

Figure S8. ^{13}C NMR spectrum of compound **1d**.

Figure S9. ^{1}H NMR spectrum of compound **1e.**

Figure S10. ^{13}C NMR spectrum of compound **1e**.

Figure S11. ^{1}H NMR spectrum of compound **1f.**

Figure S12. ^{13}C NMR spectrum of compound **1f**.

Figure S13. ^{1}H NMR spectrum of compound **1g.**

Figure S14. ^{13}C NMR spectrum of compound **1g**.

Figure S15. ^{1}H NMR spectrum of compound **1h.**

Figure S16. ^{13}C NMR spectrum of compound **1h**.

Figure S17. ^{1}H NMR spectrum of compound **2a.**

Figure S18. ^{13}C NMR spectrum of compound **2a**.

Figure S19. ^{1}H NMR spectrum of compound **2b.**

Figure S20. ^{13}C NMR spectrum of compound **2b**.

Figure S21. ^{1}H NMR spectrum of compound **2c.**

Figure S22. ^{13}C NMR spectrum of compound **2c**.

Figure S23. ^{1}H NMR spectrum of compound **2d.**

Figure S24. ^{13}C NMR spectrum of compound **2d**.

Figure S25. ^{1}H NMR spectrum of compound **2e.**

Figure S26. ^{13}C NMR spectrum of compound **2e**.

Figure S27. ^{1}H NMR spectrum of compound **2f.**

Figure S28. ^{13}C NMR spectrum of compound **2f**.

Figure S29. ^{1}H NMR spectrum of compound **2g.**

Figure S30. ^{13}C NMR spectrum of compound **2g**.

Figure S31. ^{1}H NMR spectrum of compound **2h.**

Figure S32. ^{13}C NMR spectrum of compound **2h**.

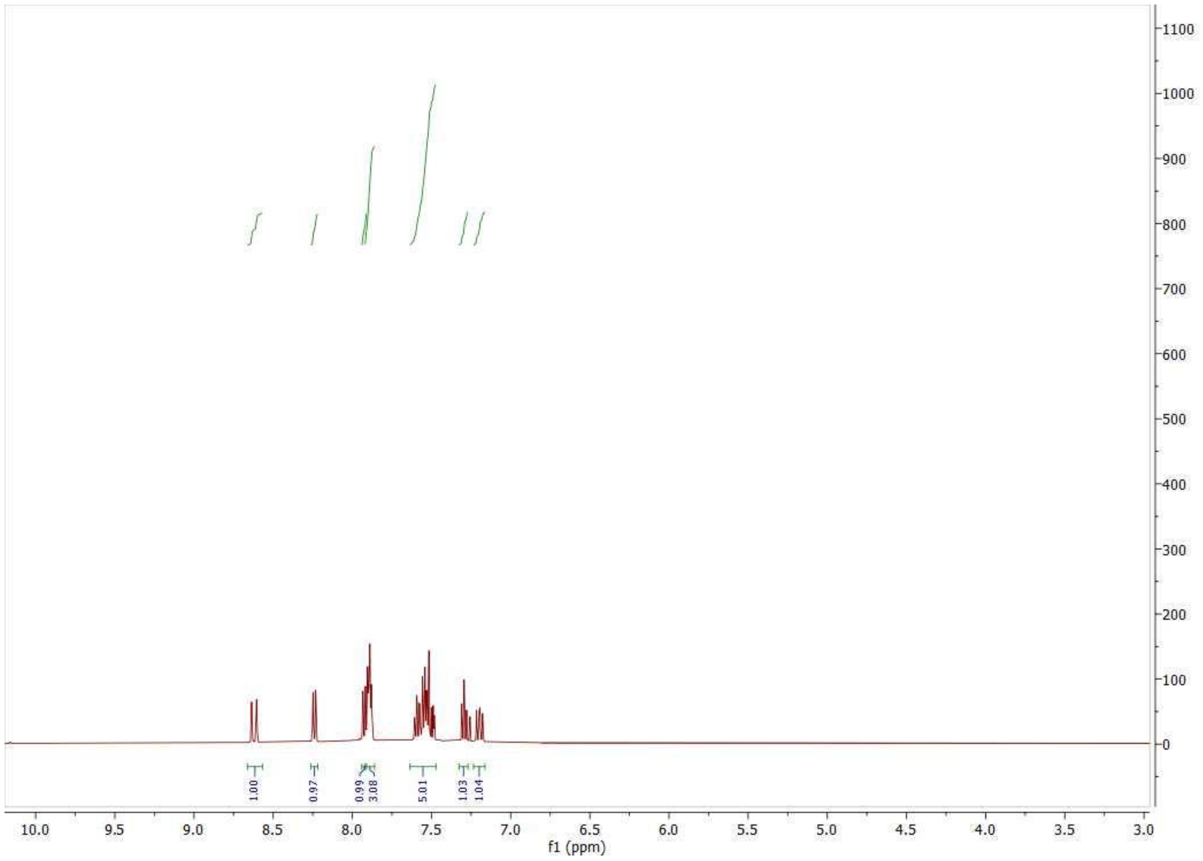

Figure S1. ^{1}H NMR spectrum of compound **1a.**

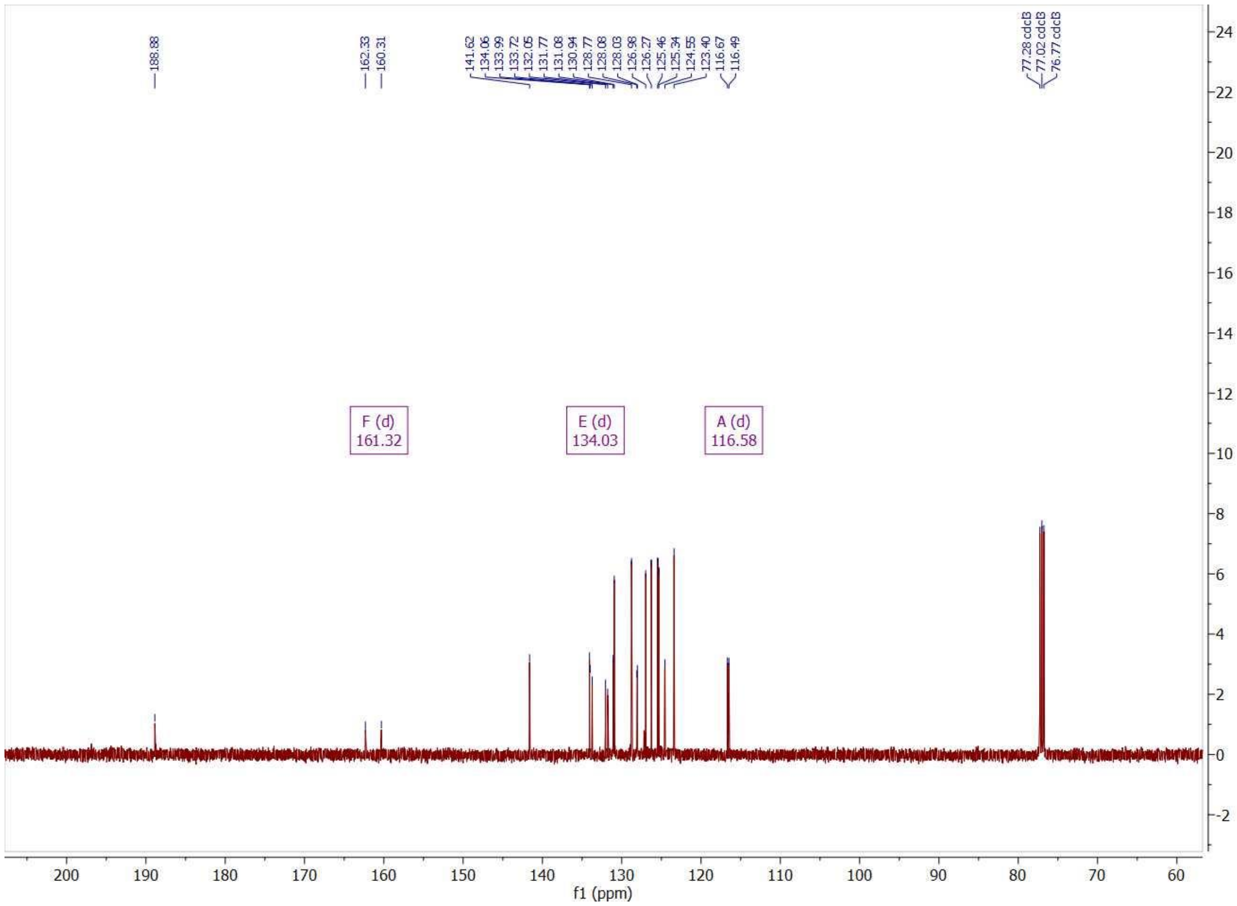

Figure S2. ^{13}C NMR spectrum of compound **1a**.

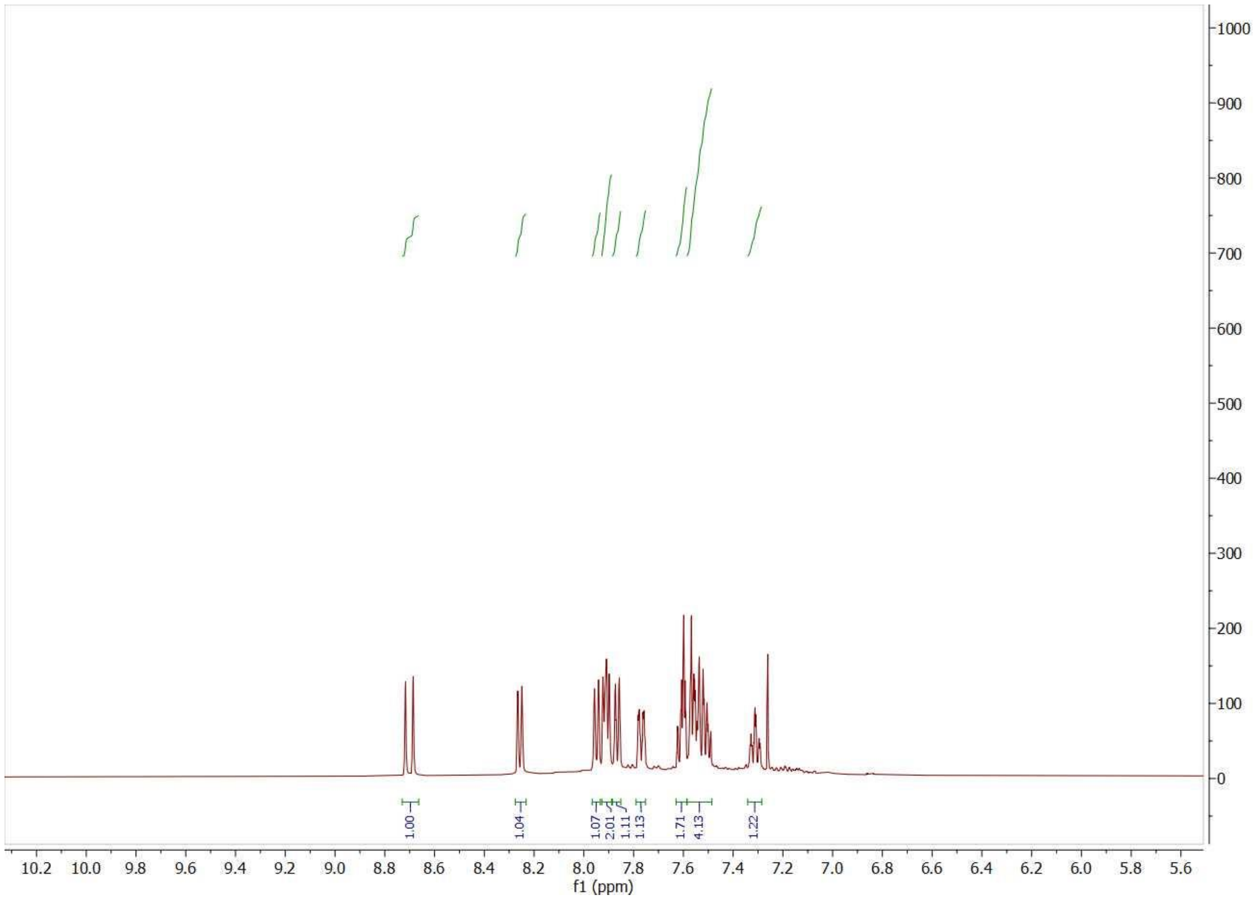

Figure S3. ^{1}H NMR spectrum of compound **1b.**

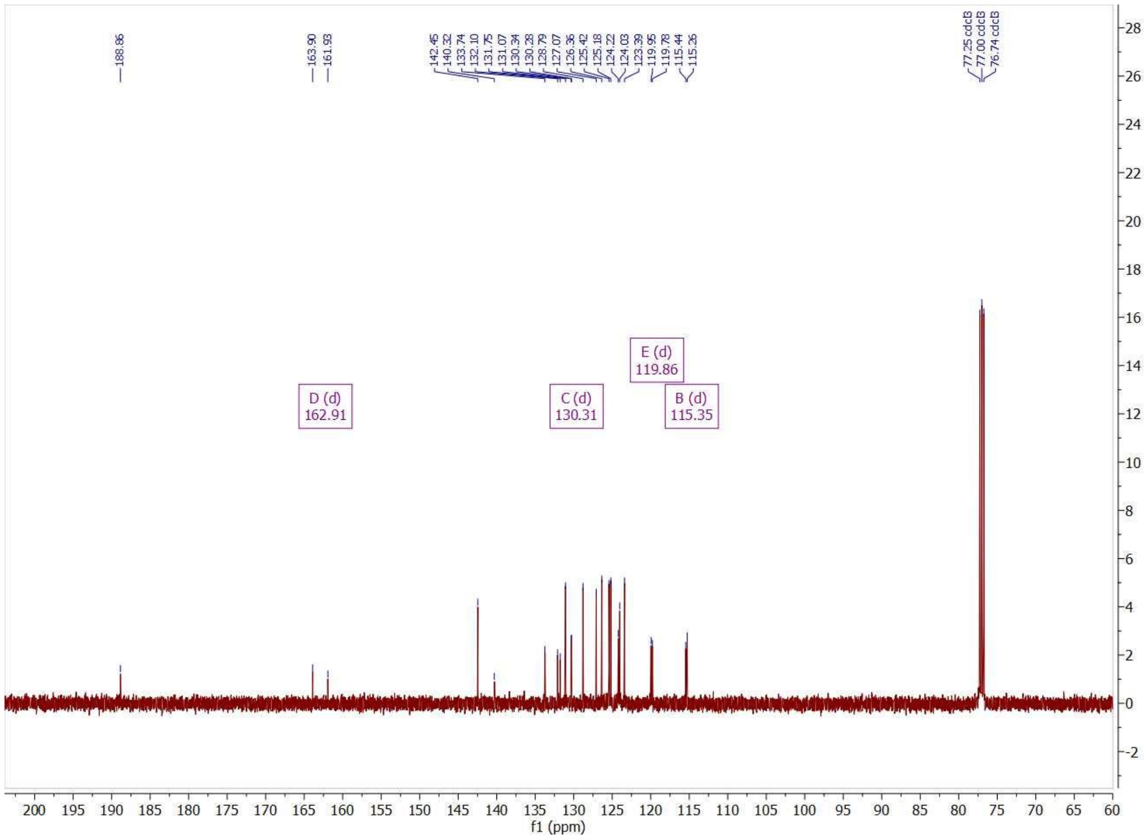

Figure S4. ^{13}C NMR spectrum of compound **1b**.

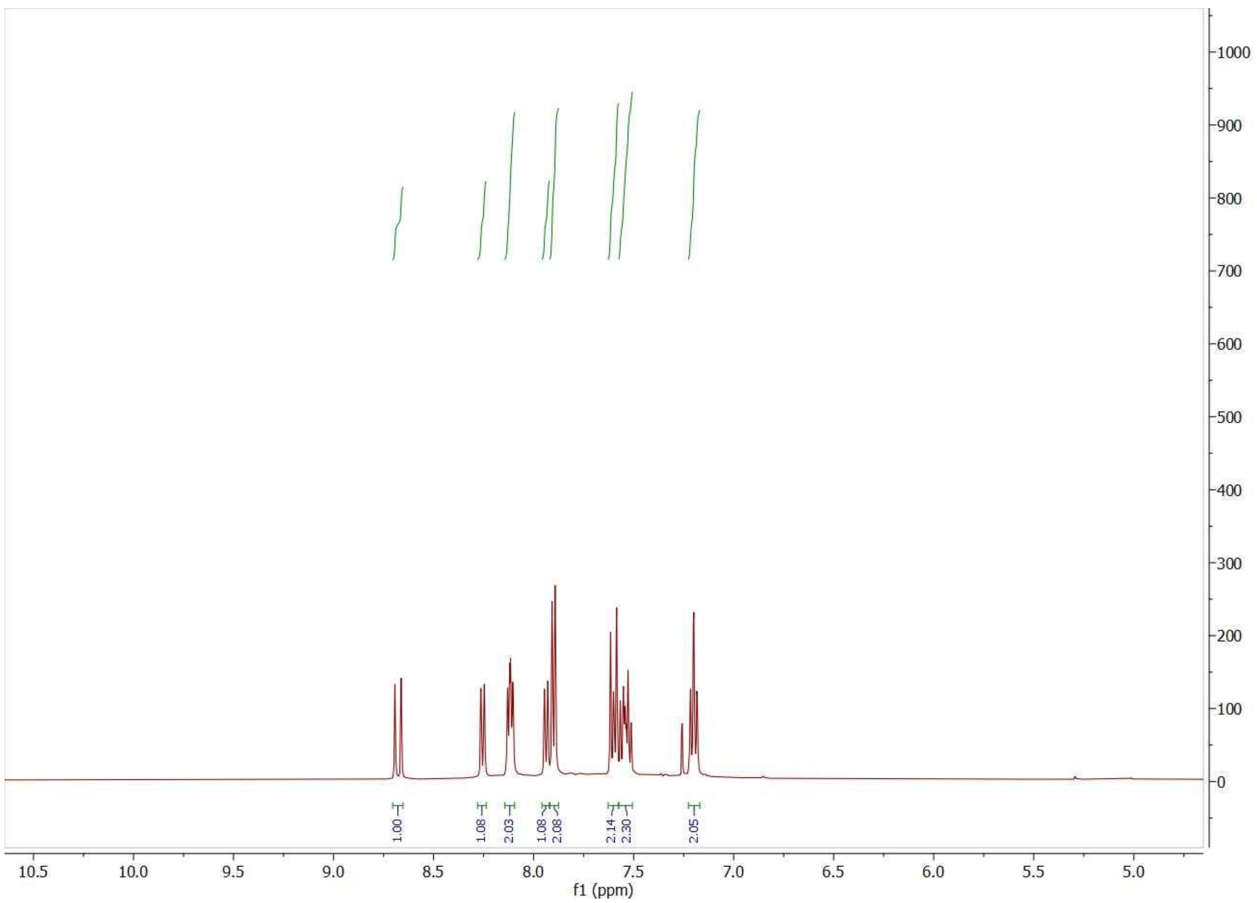

Figure S5. ^{1}H NMR spectrum of compound **1c.**

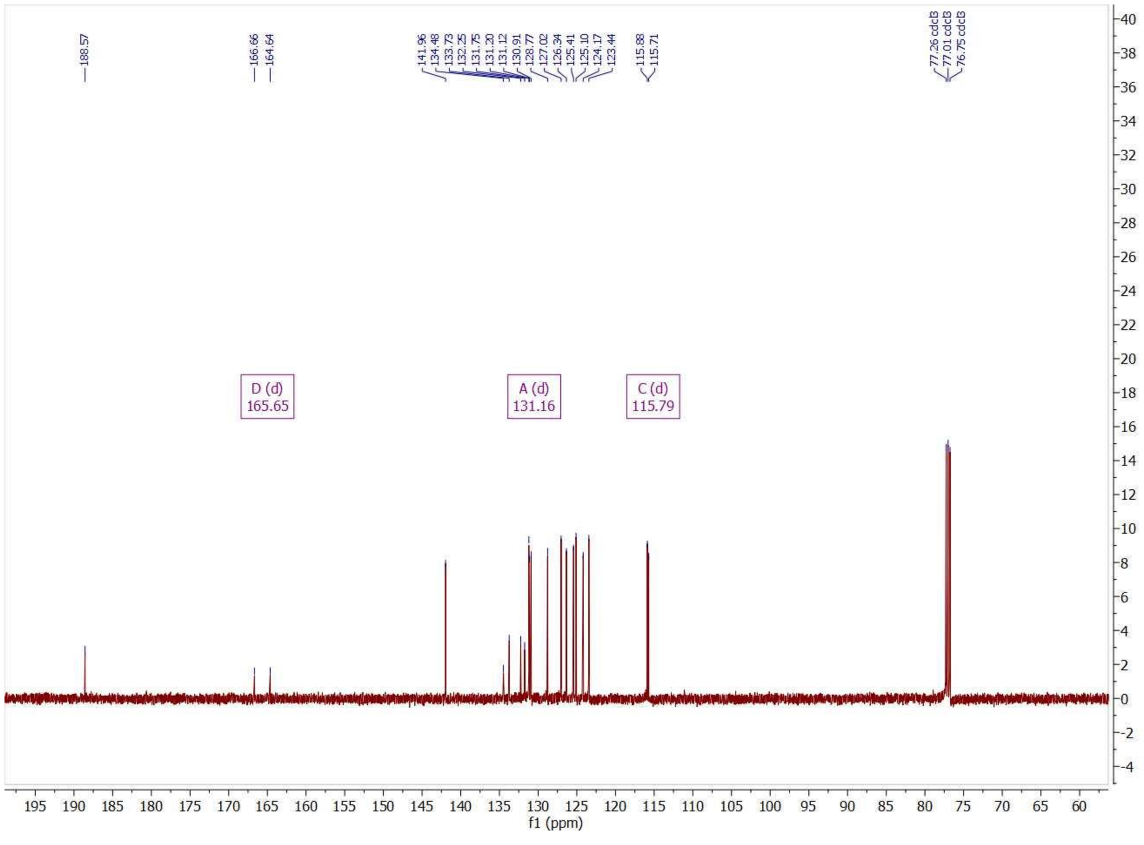

Figure S6. ^{13}C NMR spectrum of compound **1c**.

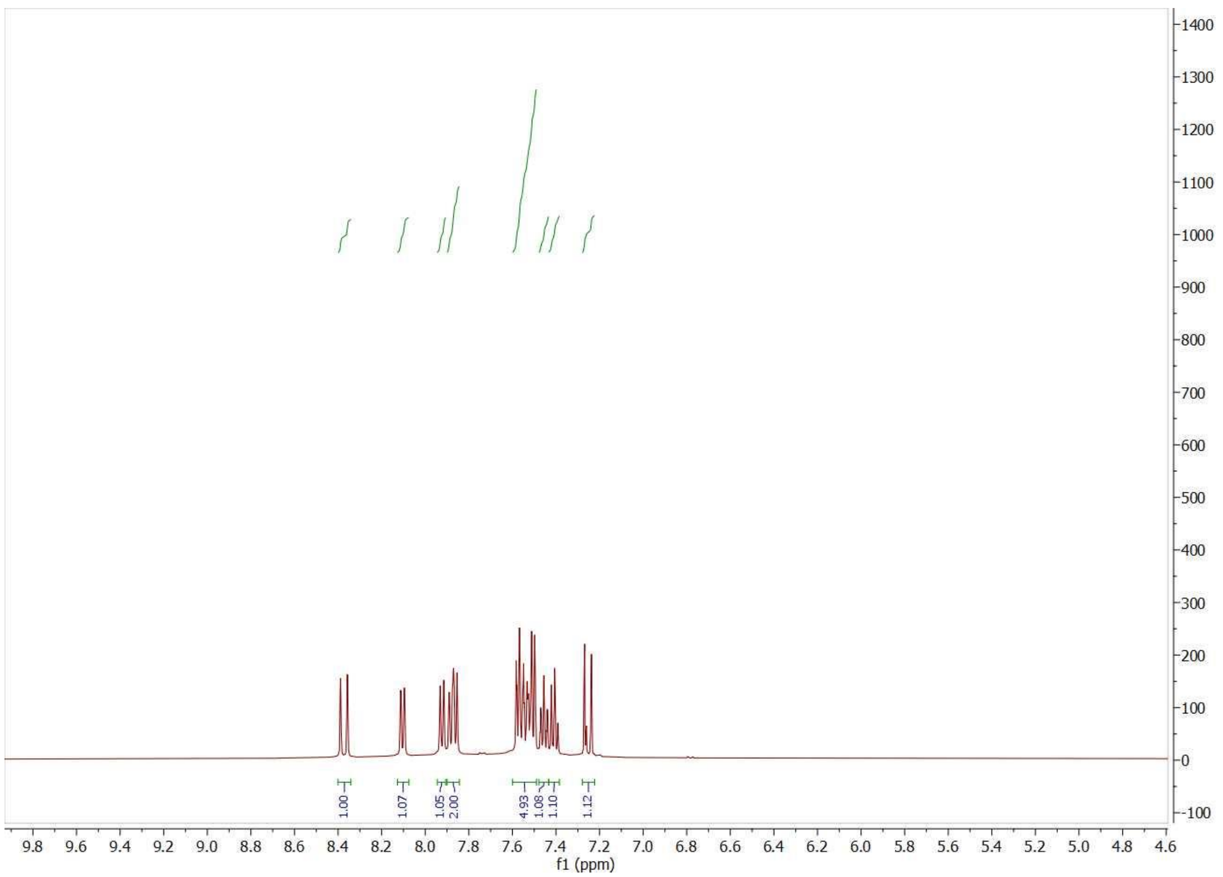

Figure S7. ^{1}H NMR spectrum of compound **1d.**

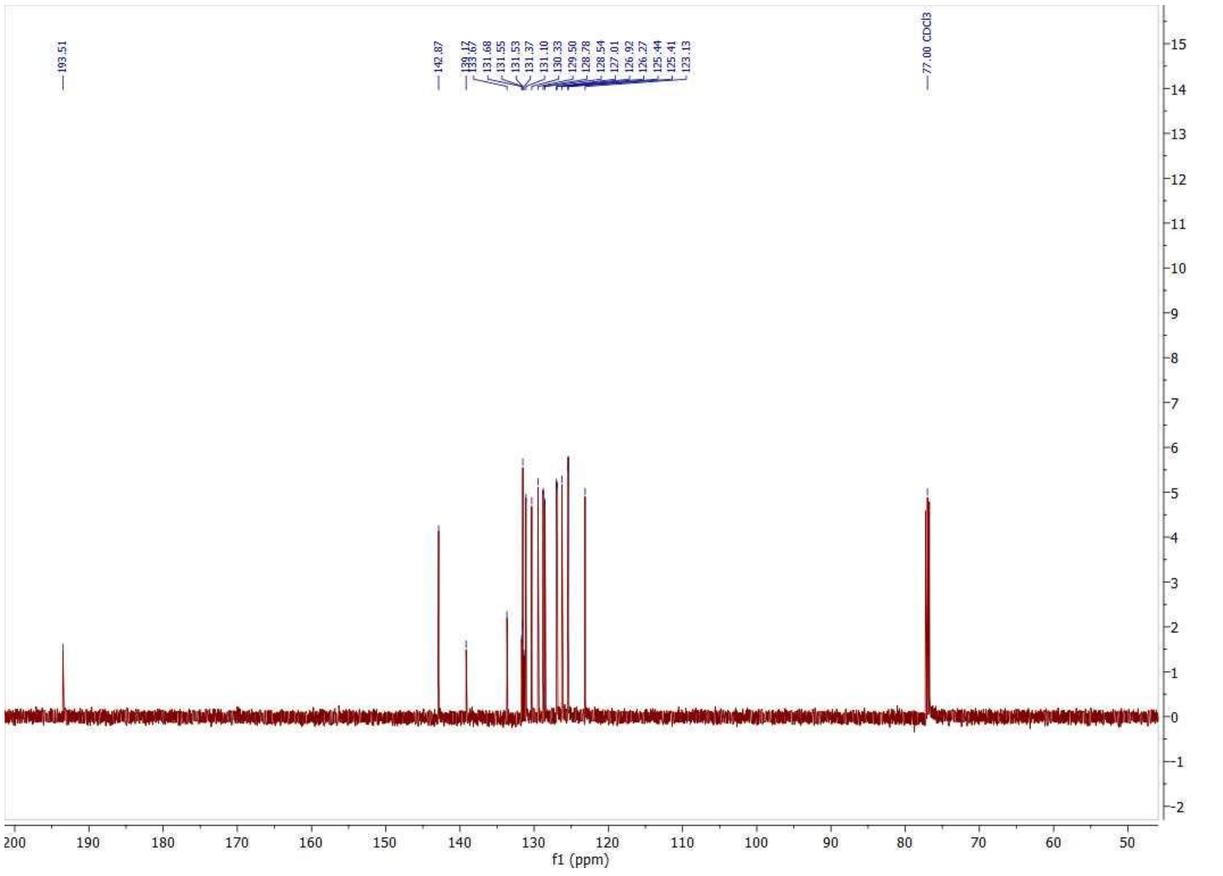

Figure S8. ^{13}C NMR spectrum of compound **1d**.

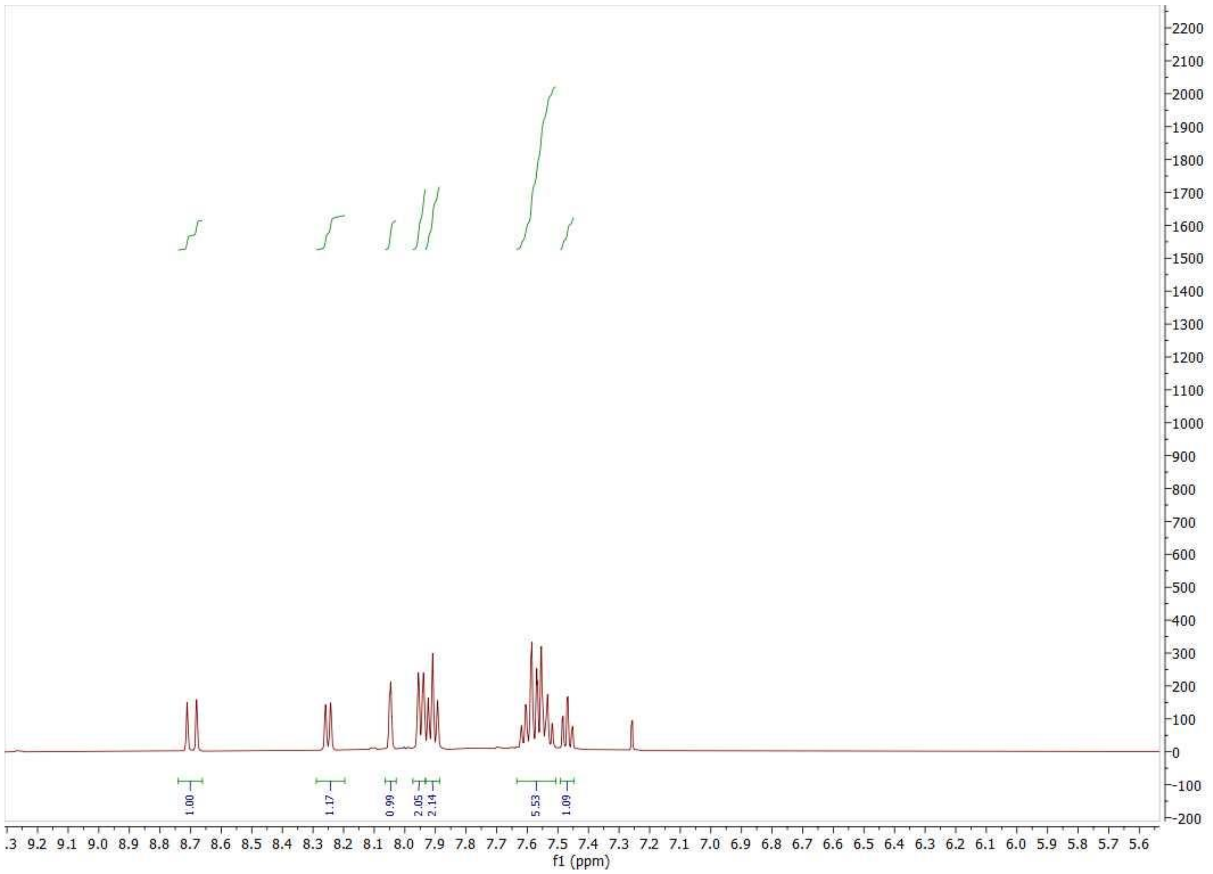

Figure S9. ^{1}H NMR spectrum of compound **1e.**

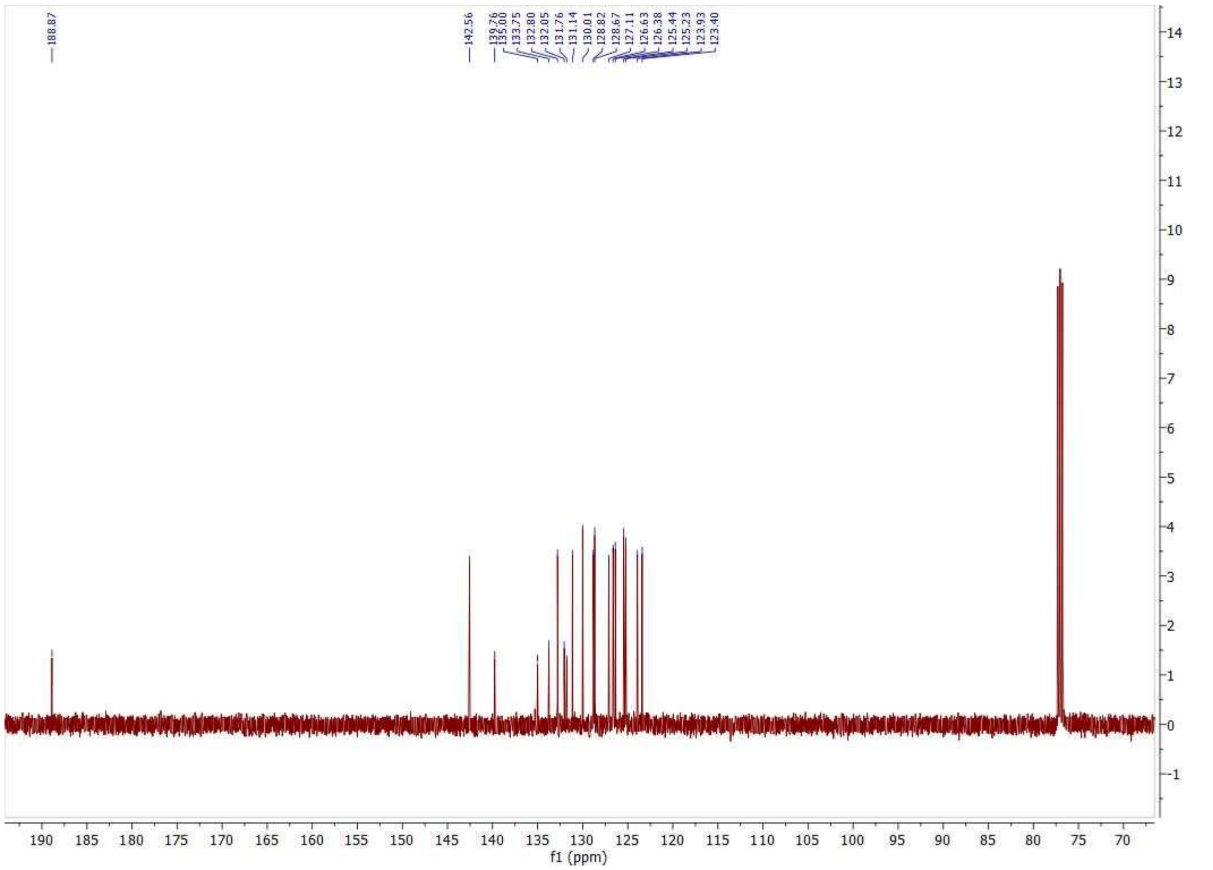

Figure S10. ^{13}C NMR spectrum of compound **1e**.

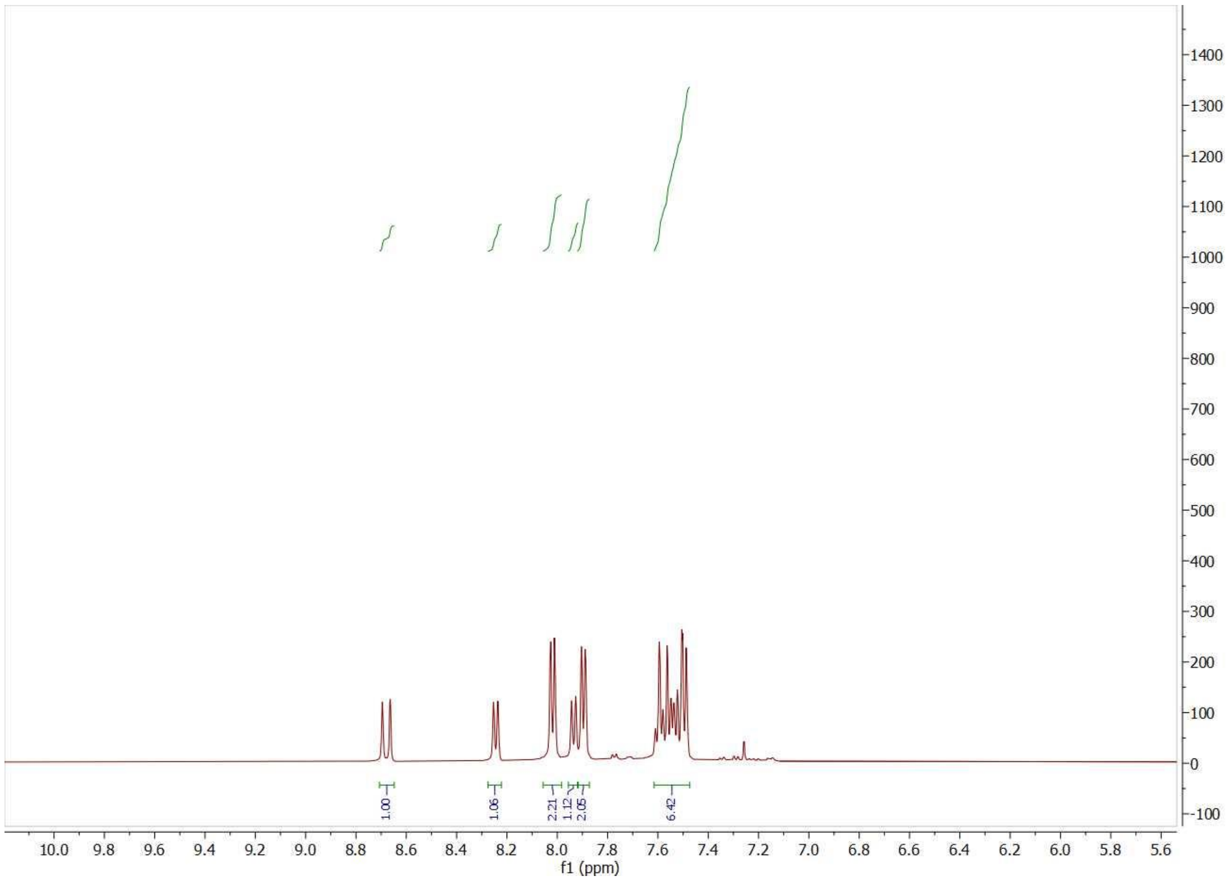

Figure S11. ^{1}H NMR spectrum of compound **1f.**

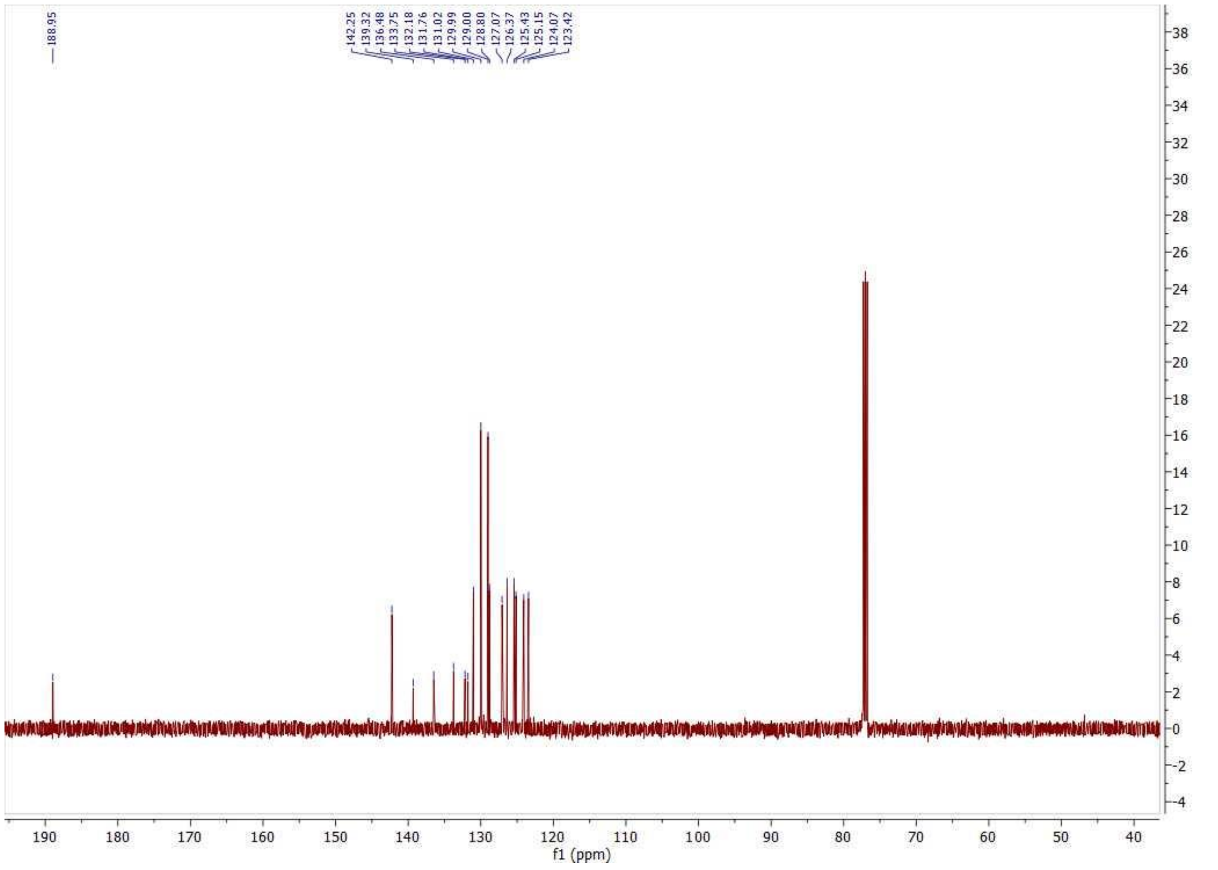

Figure S12. ^{13}C NMR spectrum of compound **1f**.

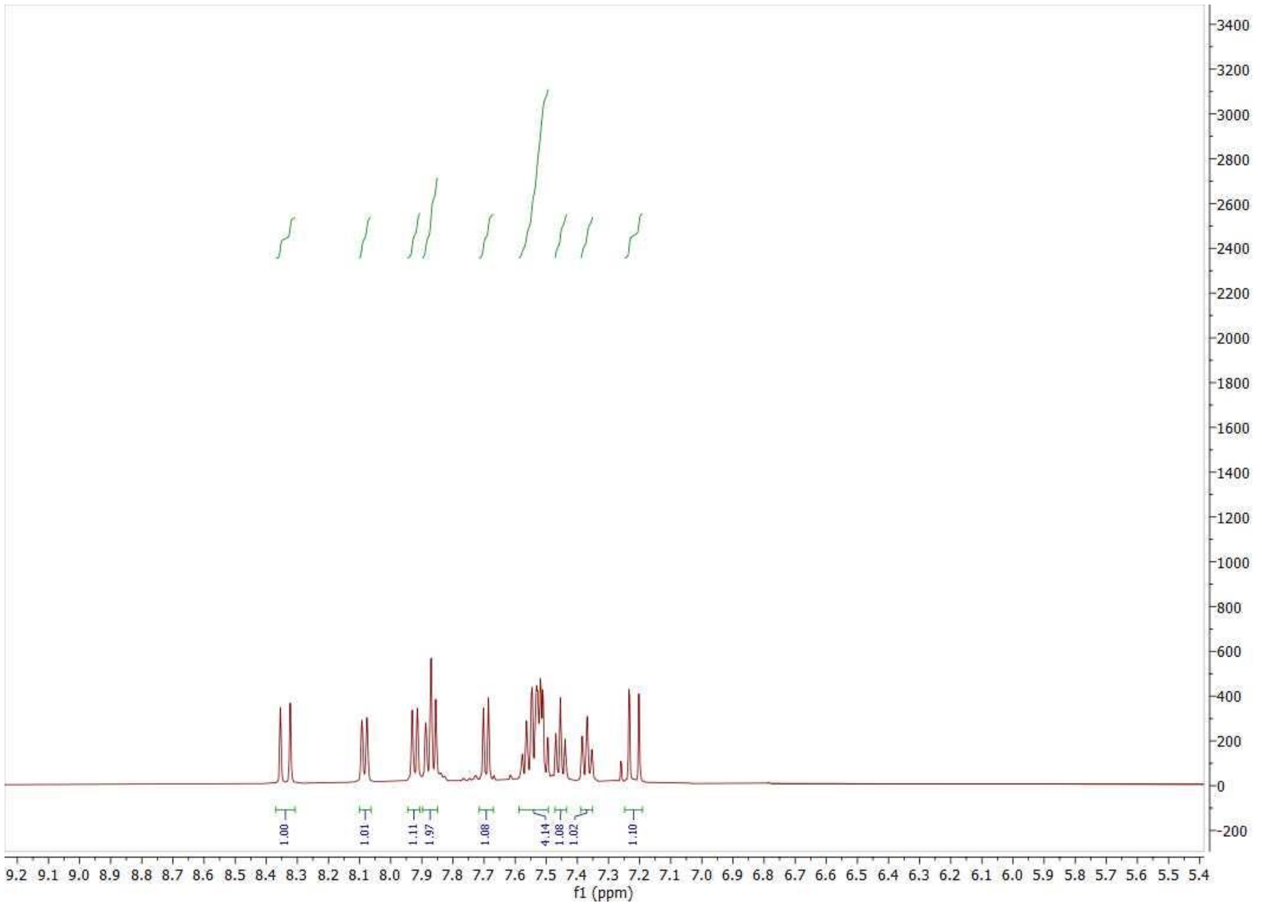

Figure S13. ^{1}H NMR spectrum of compound **1g.**

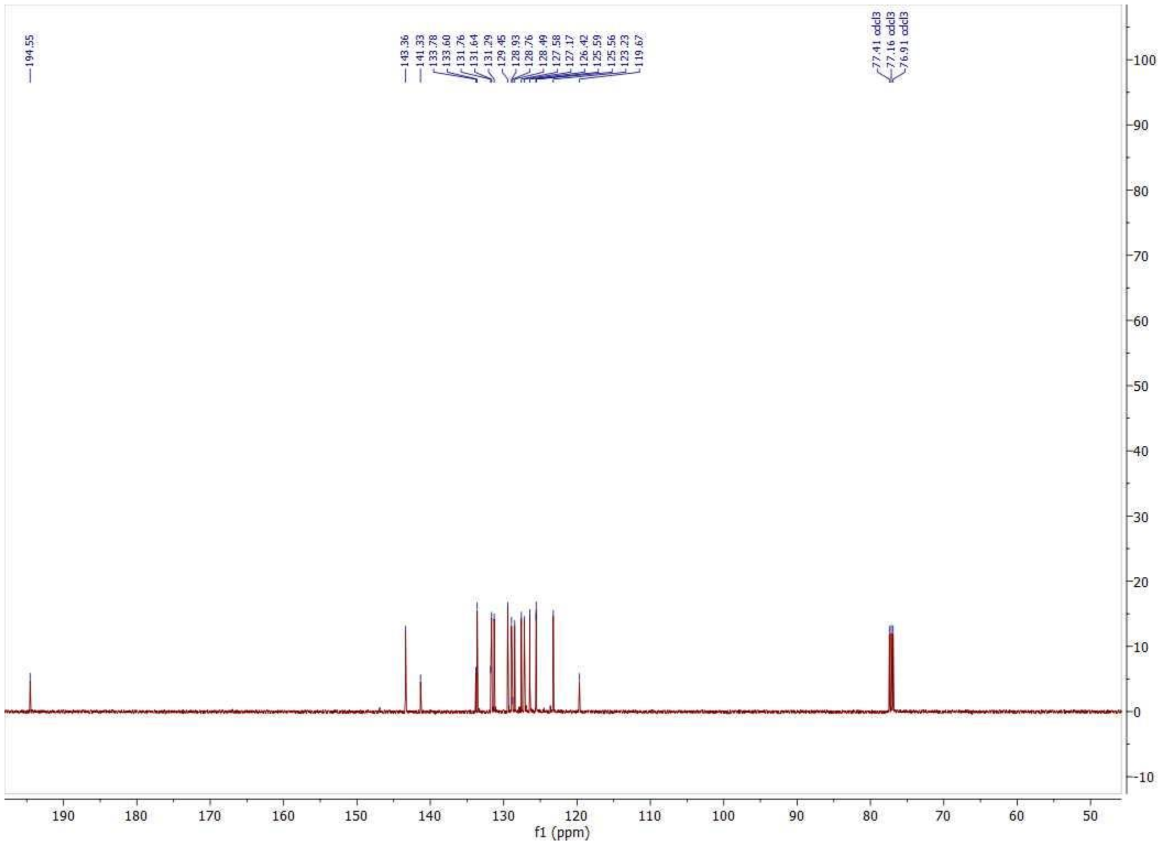

Figure S14. ^{13}C NMR spectrum of compound **1g**.

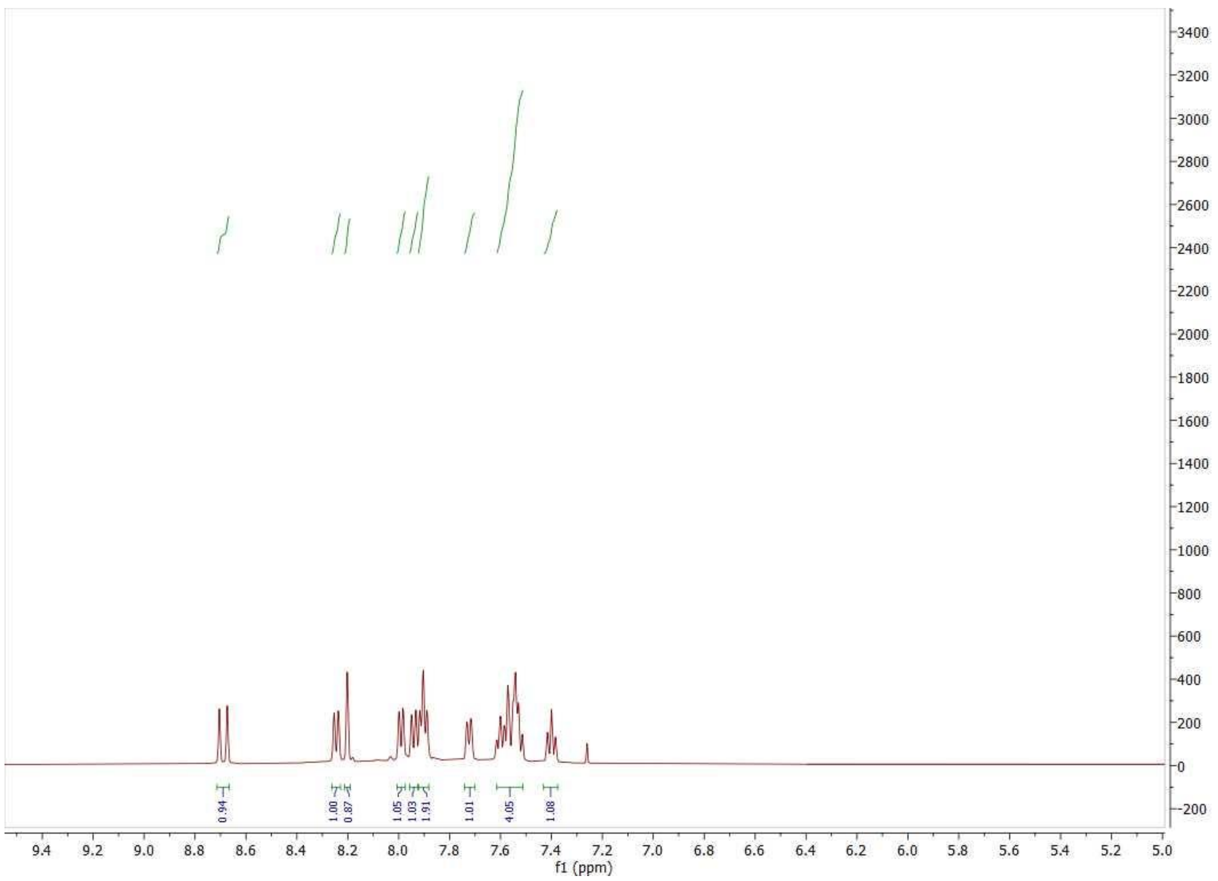

Figure S15. ^{1}H NMR spectrum of compound **1h.**

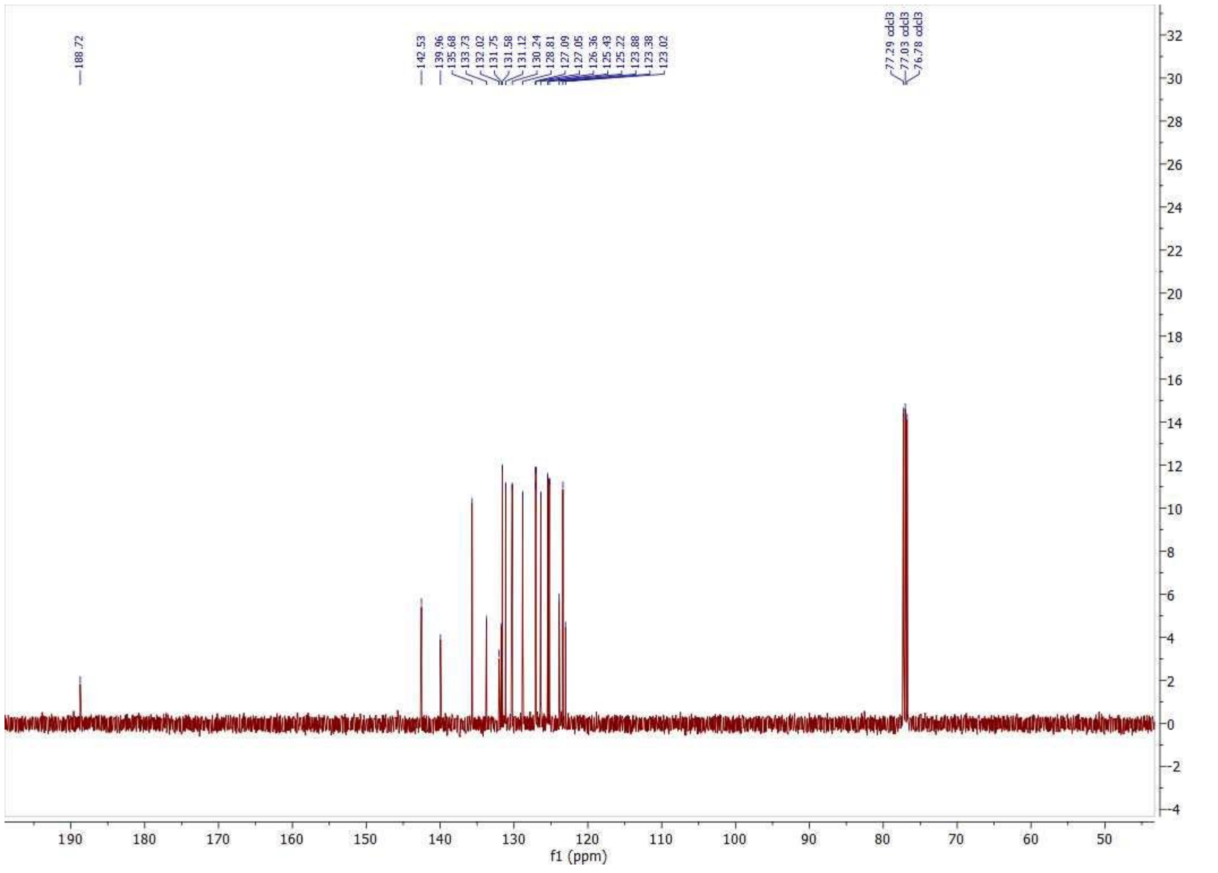

Figure S16. ^{13}C NMR spectrum of compound **1h**.

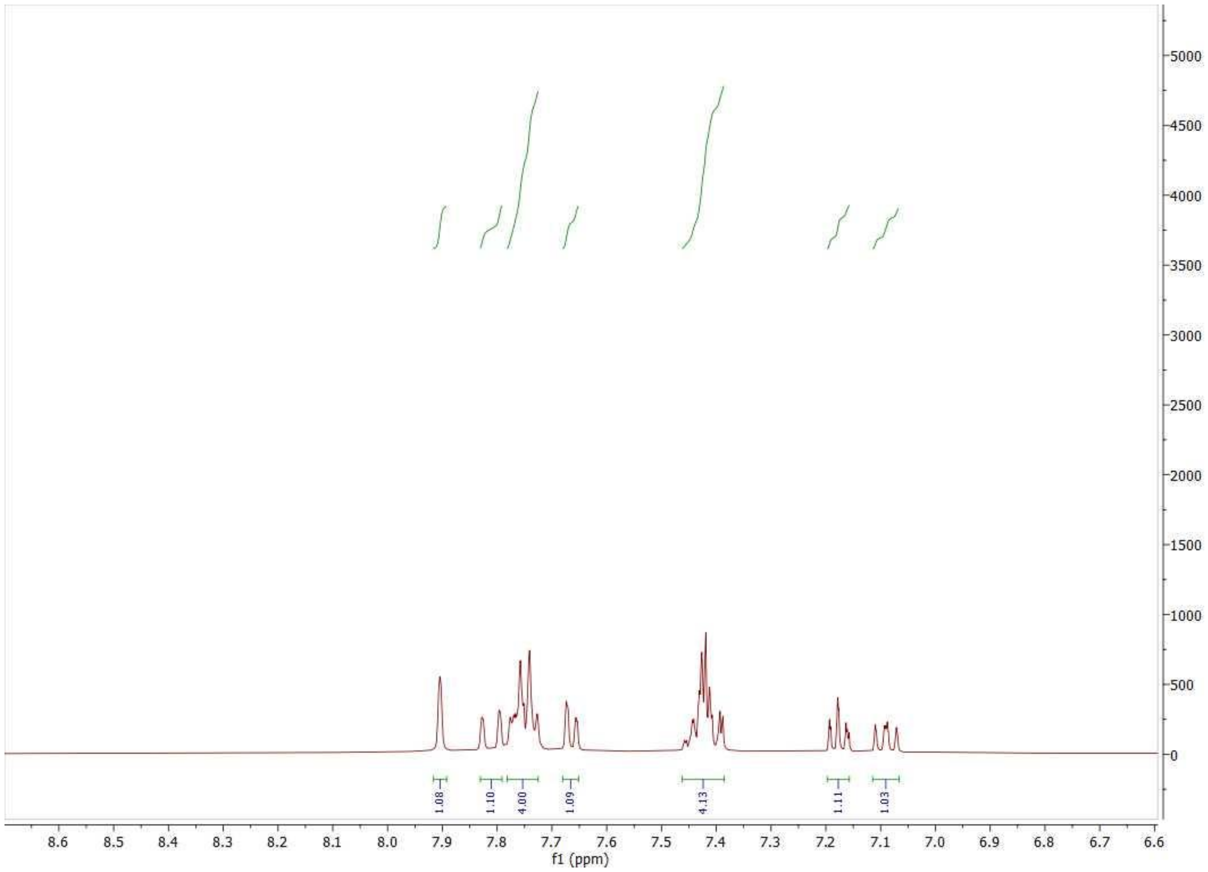

Figure S17. ^{1}H NMR spectrum of compound **2a.**

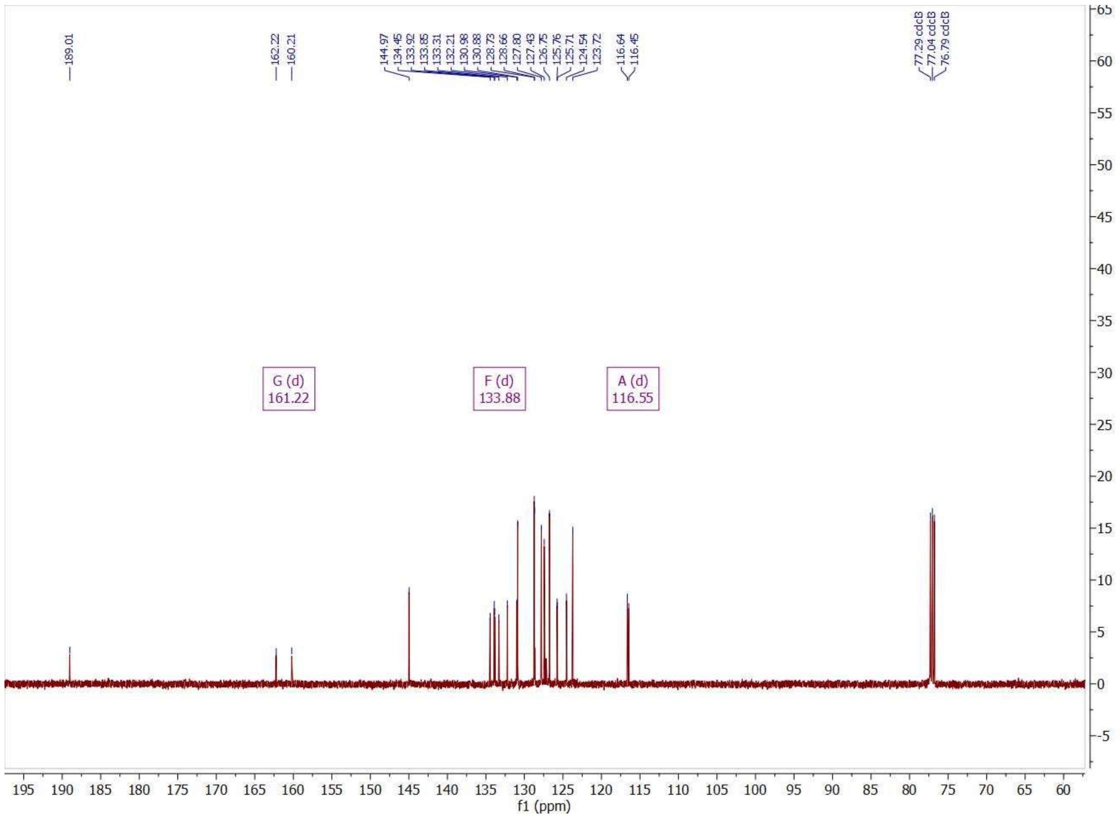

Figure S18. ^{13}C NMR spectrum of compound **2a**.

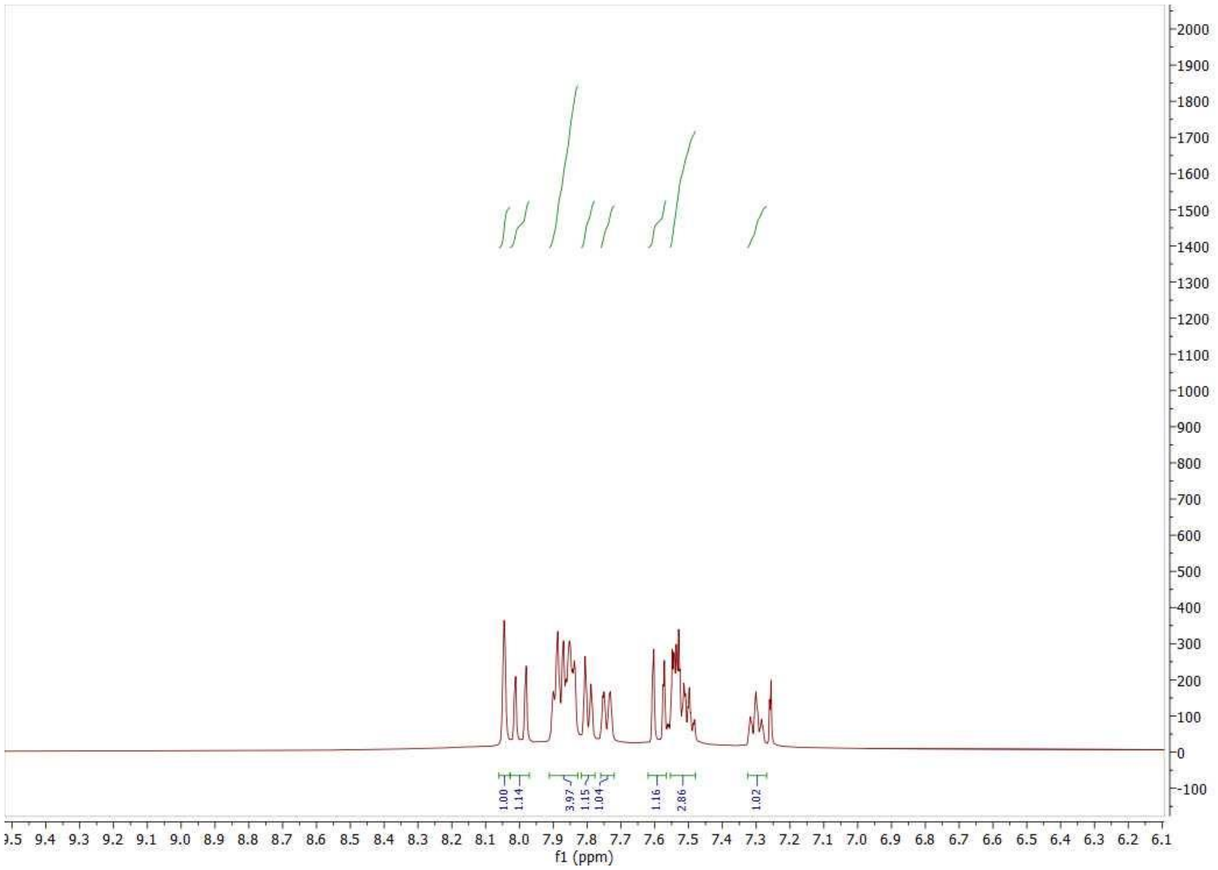

Figure S19. ^{1}H NMR spectrum of compound **2b.**

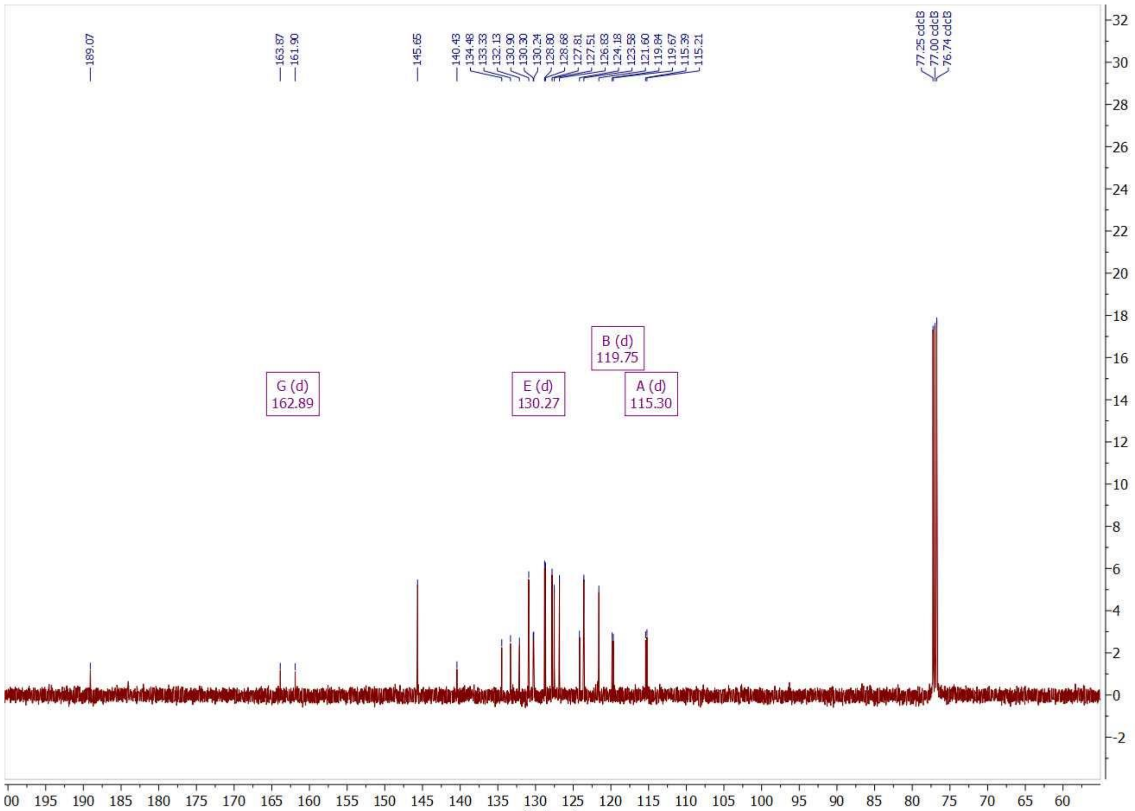

Figure S20. ^{13}C NMR spectrum of compound **2b**.

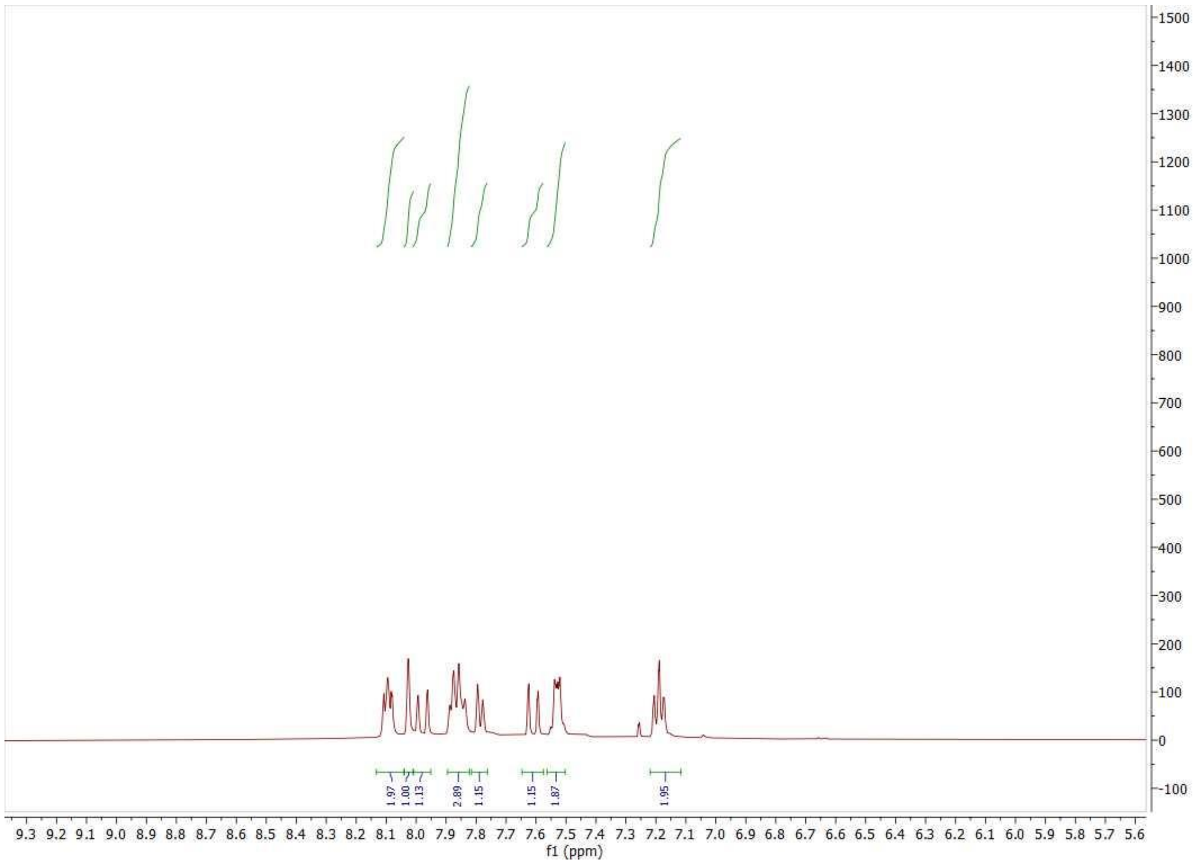

Figure S21. ^{1}H NMR spectrum of compound **2c.**

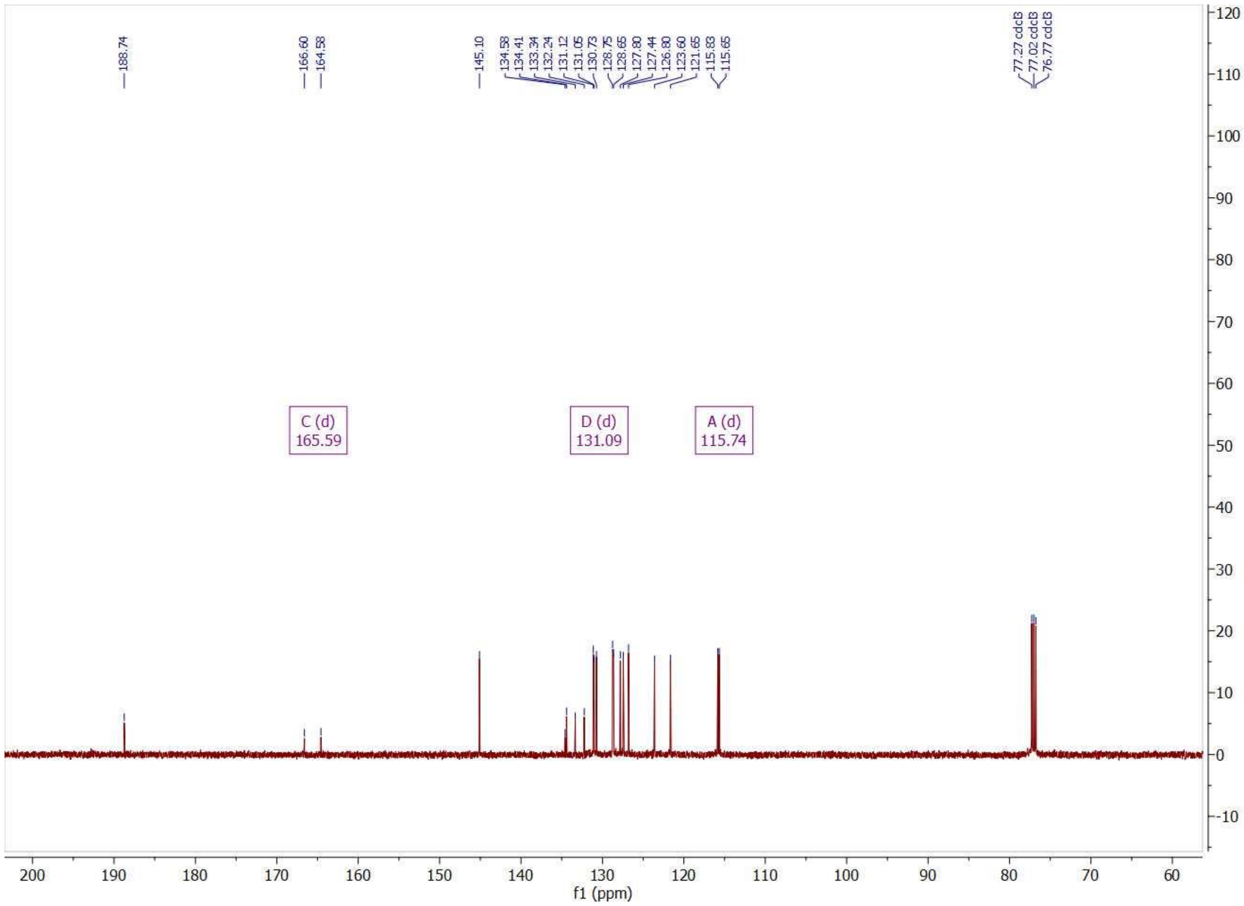

Figure S22. ^{13}C NMR spectrum of compound **2c**.

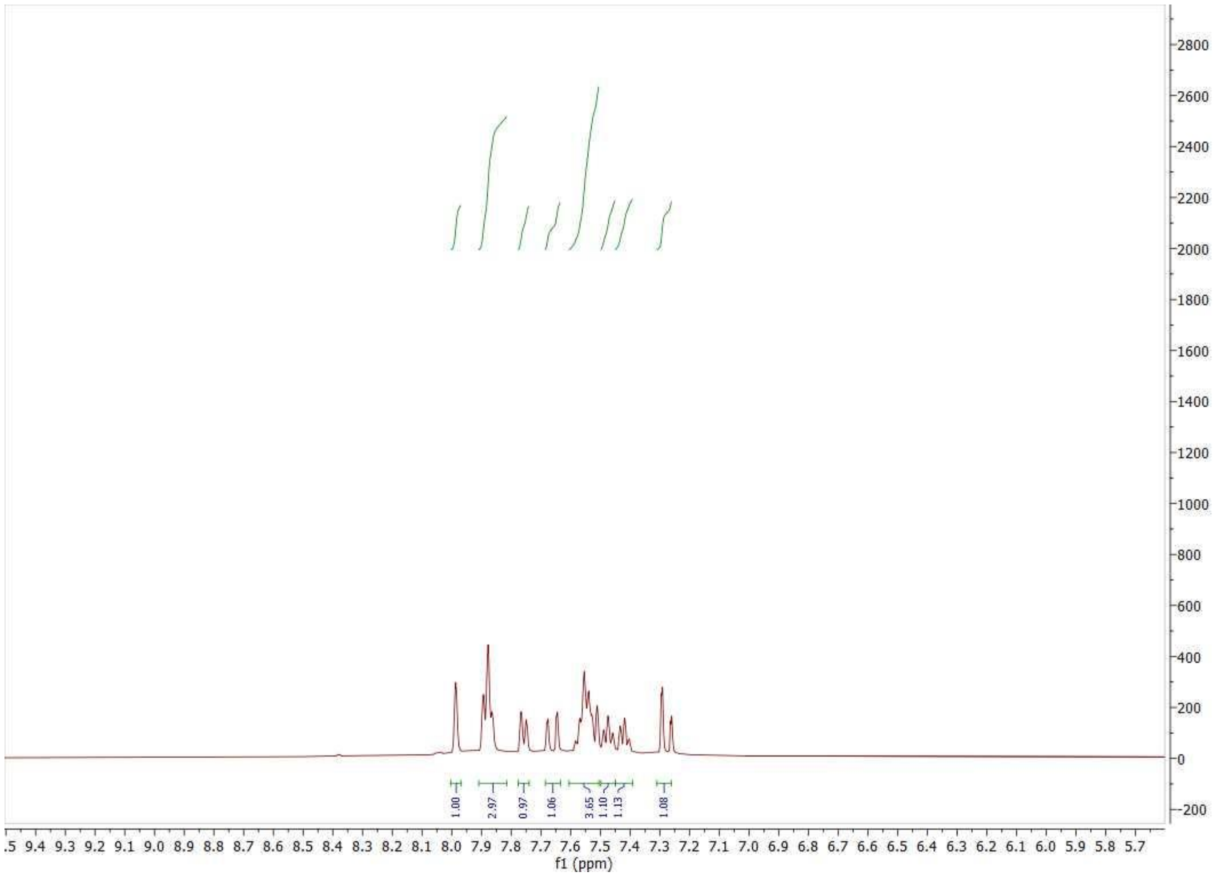

Figure S23. ^{1}H NMR spectrum of compound **2d.**

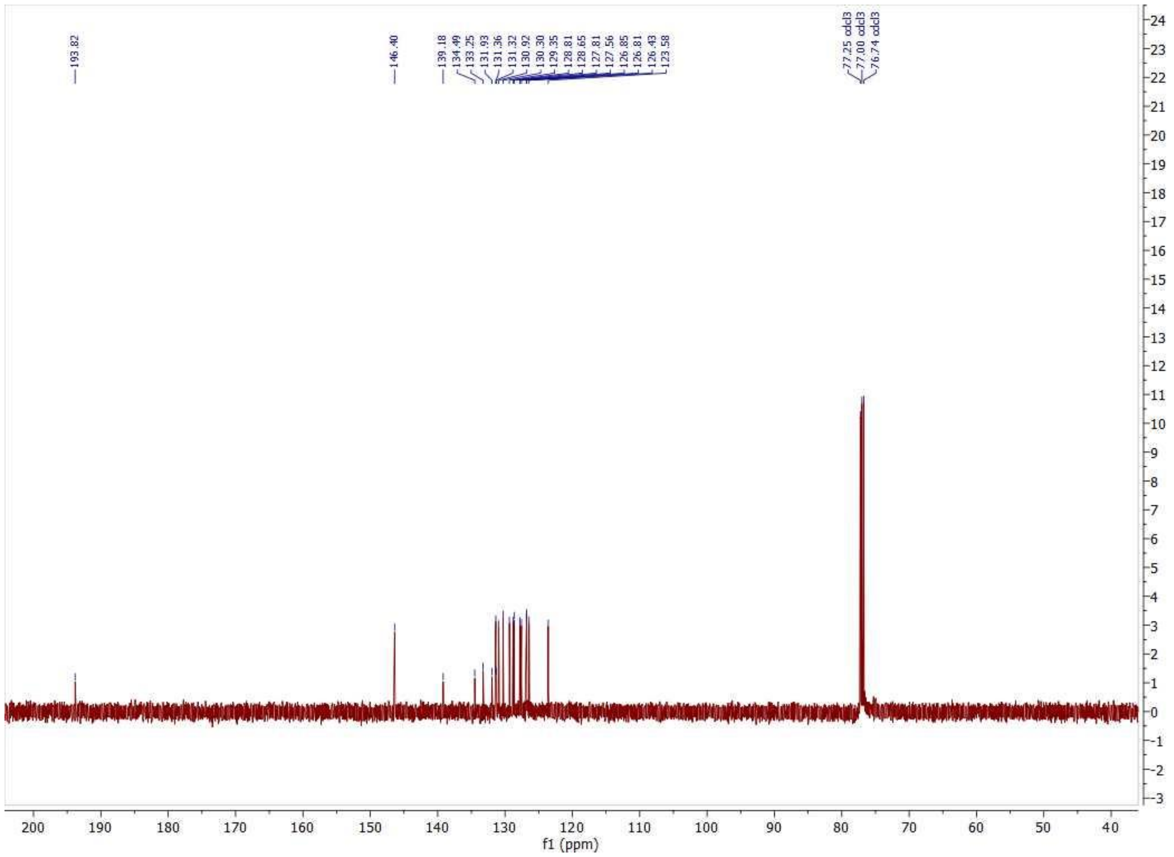

Figure S24. ^{13}C NMR spectrum of compound **2d**.

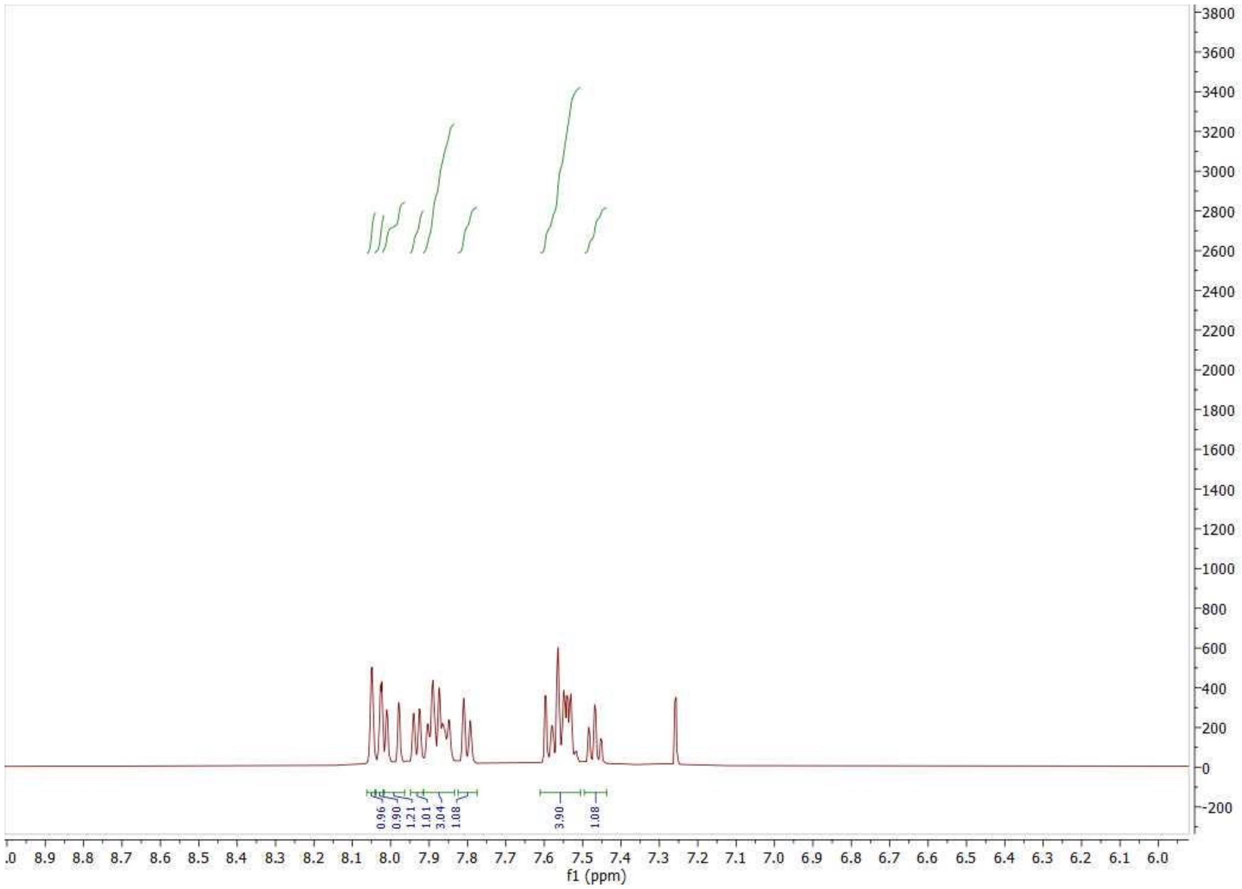

Figure S25. ^{1}H NMR spectrum of compound **2e.**

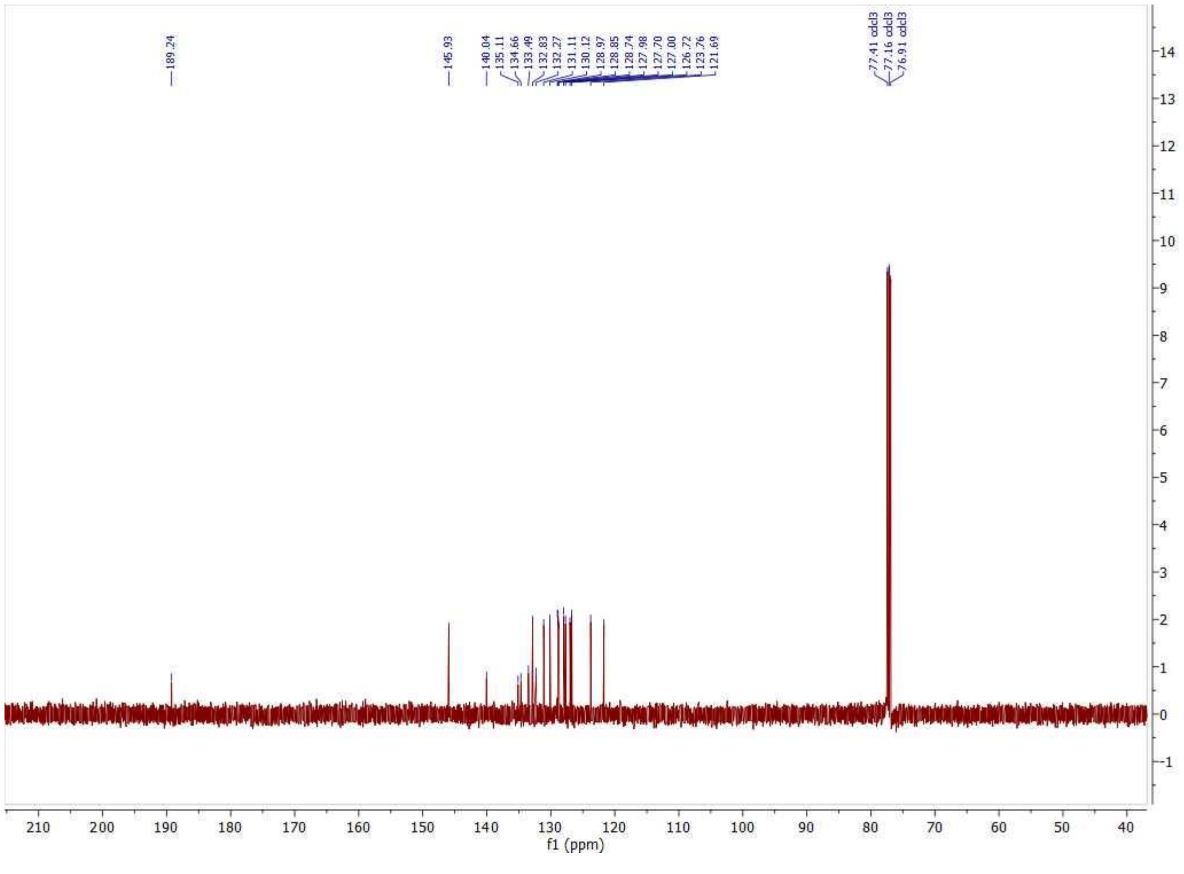

Figure S26. ^{13}C NMR spectrum of compound **2e**.

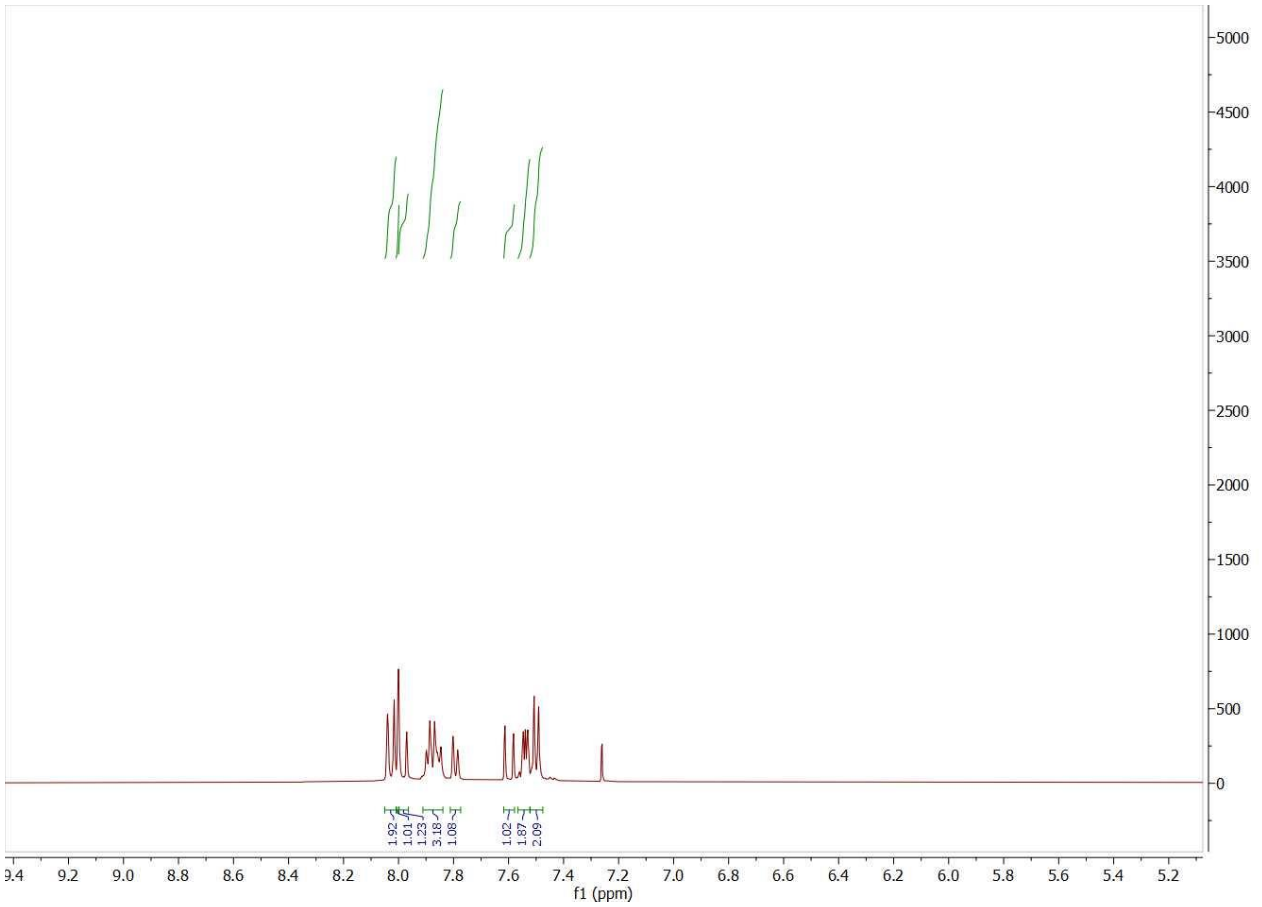

Figure S27. ^{1}H NMR spectrum of compound **2f.**

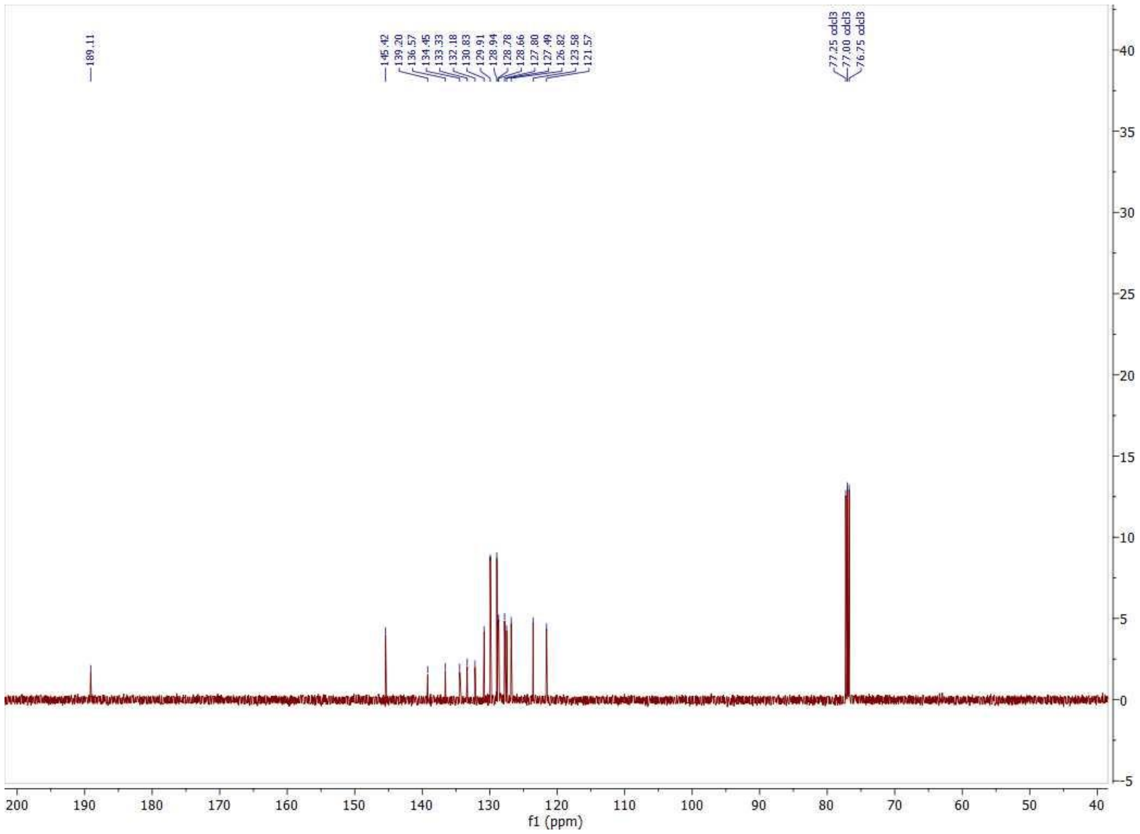

Figure S28. ^{13}C NMR spectrum of compound **2f**.

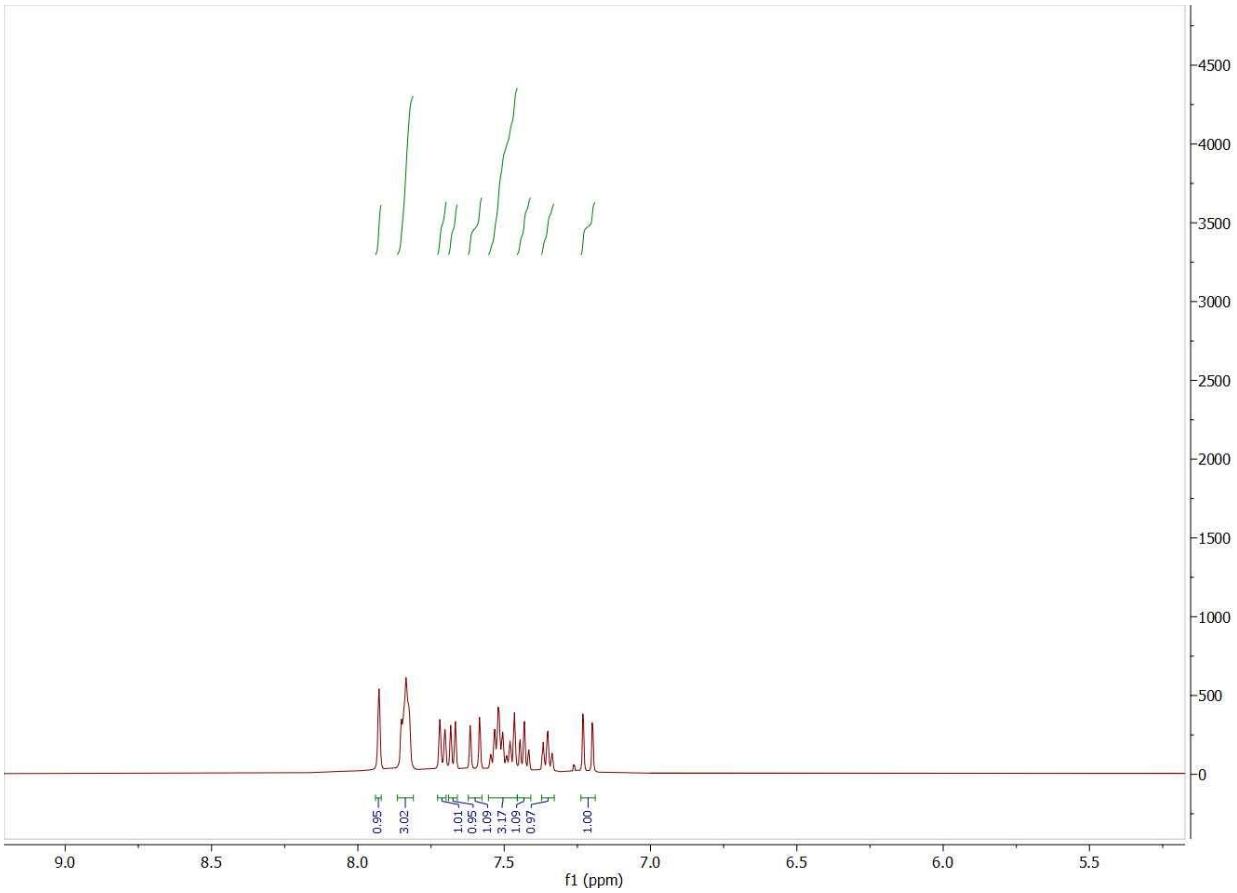

Figure S29. ^{1}H NMR spectrum of compound **2g.**

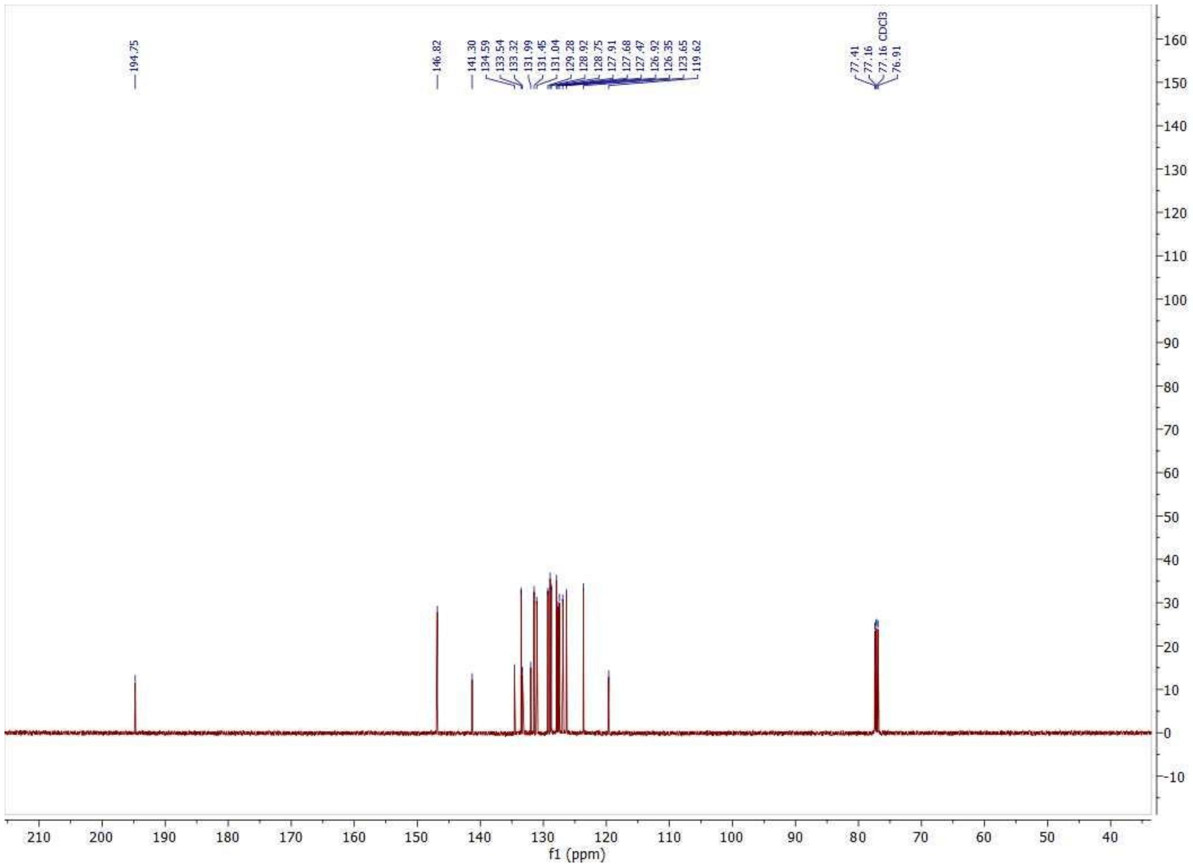

Figure S30. ^{13}C NMR spectrum of compound **2g**.

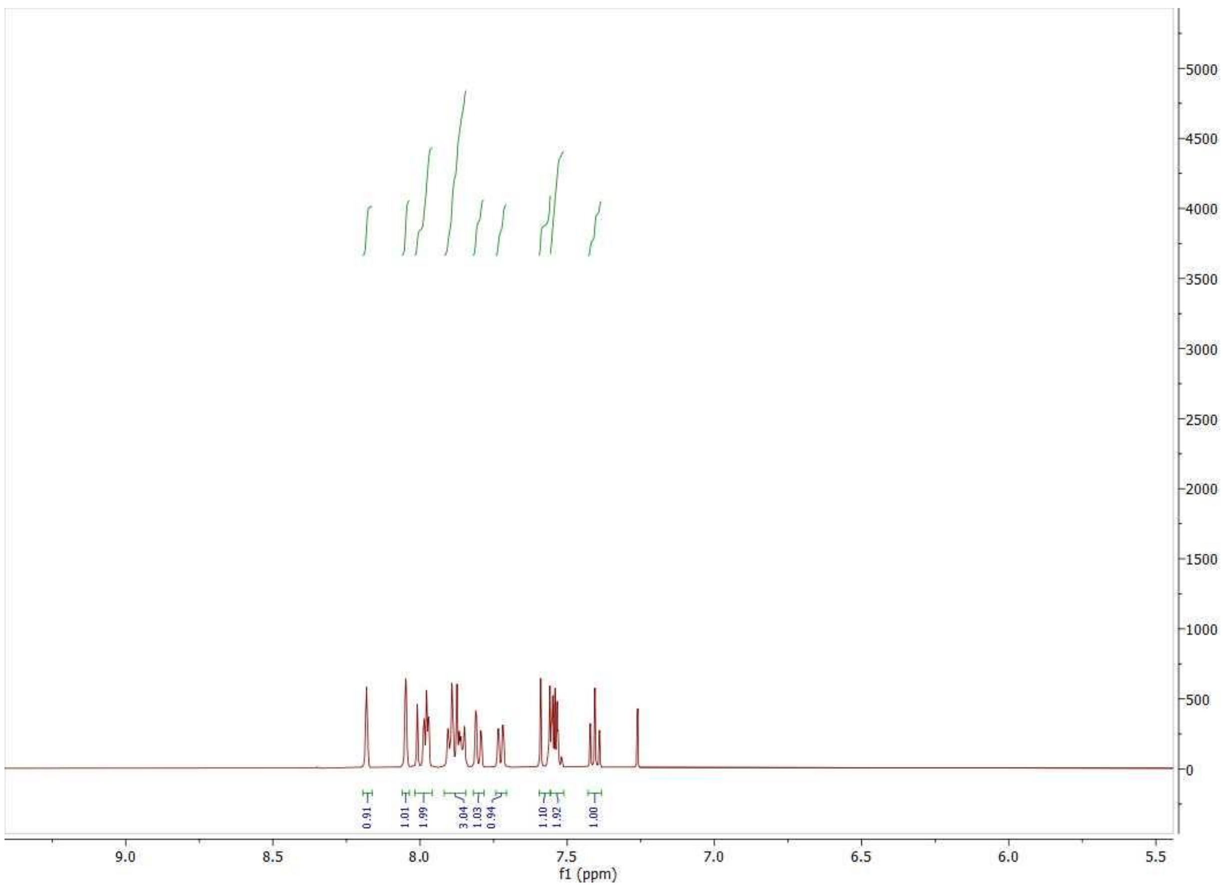

Figure S31. ^{1}H NMR spectrum of compound **2h.**

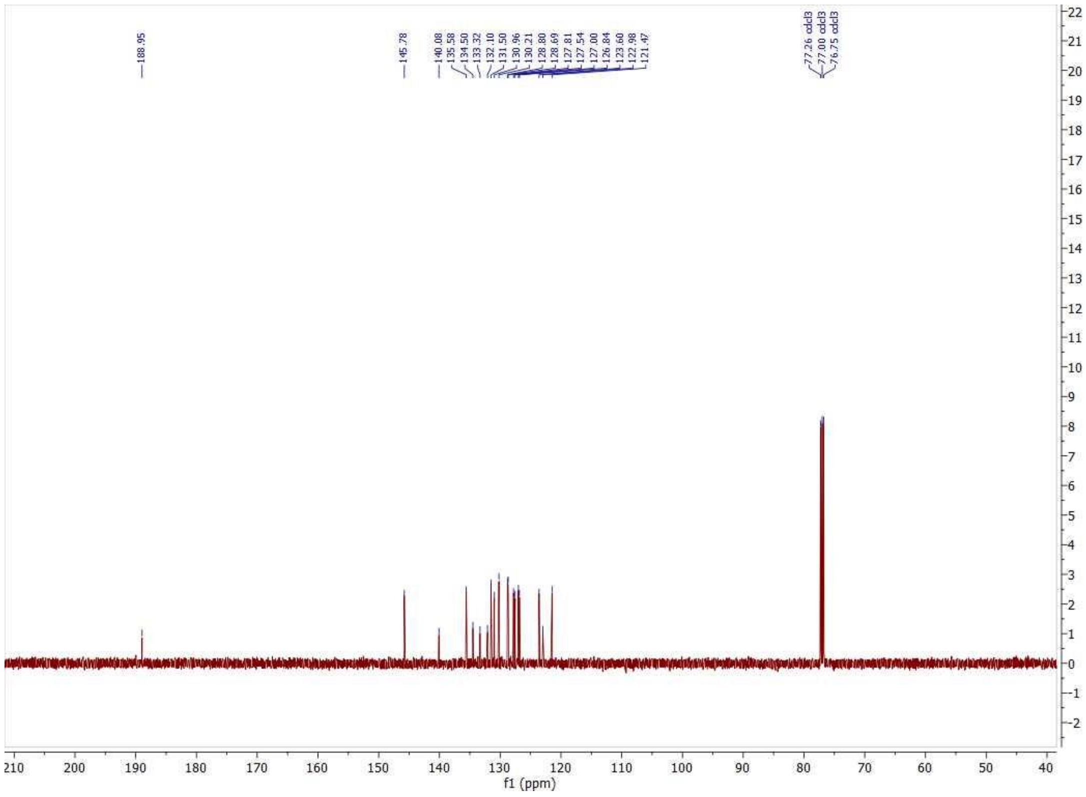

Figure S32. ^{13}C NMR spectrum of compound **2h**.
